# Inhibiting quinolone biosynthesis of *Burkholderia*†

**DOI:** 10.1039/d0sc06167k

**Published:** 2021-03-26

**Authors:** Michaela Prothiwa, Verena Filz, Sebastian Oehler, Thomas Böttcher

**Affiliations:** Department of Chemistry, Konstanz Research School Chemical Biology, University of Konstanz 78457 Konstanz Germany; Faculty of Chemistry, Department of Biological Chemistry & Centre for Microbiology and Environmental Systems Science, Division of Microbial Ecology, University of Vienna 1090 Vienna Austria Thomas.Boettcher@univie.ac.at

## Abstract

2-Alkylquinolones are important signalling molecules of *Burkholderia* species. We developed a substrate-based chemical probe against the central quinolone biosynthesis enzyme HmqD and applied it in competitive profiling experiments to discover the first known HmqD inhibitors. The most potent inhibitors quantitatively blocked quinolone production in *Burkholderia* cultures with single-digit micromolar efficacy.

## Abstract 2

Competitive profiling with a chemical probe against the central quinolone biosynthesis enzyme HmqD results in inhibitors that quantitatively block quinolone production in *Burkholderia*.

## Graphical abstract

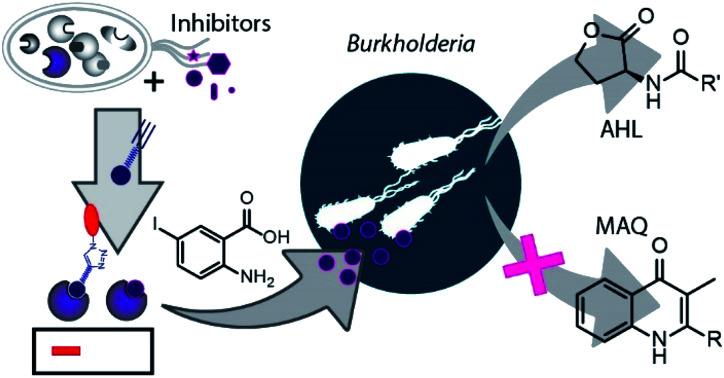

2-Alkylquinolones (AQs) and their derivatives are important bacterial metabolites. Over 50 different AQs have been reported in *Pseudomonas aeruginosa*^1^ with major roles for virulence control *via* quorum sensing,^2,3^ interspecies competition,^4,5^ and bacteria–host interactions.^6^ Homologous biosynthetic gene clusters also exist in species of the genus *Burkholderia*^7^ producing unique methylated quinolone congeners.^8,9^ These 3-methyl-2-alkylquinolones (MAQs)‡‡3-Methyl-2-alkylquinolones (MAQs) have been also termed HMAQs referring to their tautomeric 4-hydroxy-3-methyl-2-alkyl-4(1*H*)-quinolone form. To be consistent with the nomenclature of 2-alkyl-4(1*H*)-quinolones (AQs), we chose the abbreviation MAQs and MAQNOs for the corresponding *N*-oxides. are non-classical quorum sensing signals in *Burkholderia* and have been implicated in regulating homoserine lactone quorum signalling.^10^

The genus *Burkholderia* comprises important pathogens such as *B*. *pseudomallei*, the causative agent of melioidosis^11^ and species of the *B*. *cepacia* complex including *B*. *ambifaria* and *B*. *multivorans* which are responsible for severe infections in cystic fibrosis (CF) patients.^12^ MAQ production was only reported in clinical strains of *B*. *ambifaria* and was absent in virulence attenuated variants as well as in environmental isolates.^13^ This may suggest potential roles of these quinolones in pathogenicity. Customized inhibitors of quinolone biosynthesis could thus provide valuable tools for dissecting hitherto unknown functions of quinolones in *Burkholderia*. We have recently described a live-cell profiling strategy with electrophilic chemical probes that led to highly effective inhibitors of quinolone biosynthesis of *P*. *aeruginosa*.^14^ We now take this strategy one step further to interrogate the function of HmqD, the postulated homologous quinolone synthetase of *Burkholderia* and discovered the first inhibitors of the quinolone production in live cells of this genus.

While the *pqsA-E* quinolone gene cluster of *P*. *aeruginosa* has been investigated in great detail over last years, species of the genus *Burkholderia* presumably use the gene products of hmqA-G for the biosynthesis of MAQs (Fig. 1a). The genes of the hmq cluster have been characterized by their homologies with genes of the pqs cluster and several of them also by analysis of knockout mutants.^8^ A central step in the biosynthesis of the quinolones of *P*. *aeruginosa* is the production of a coenzyme A-activated 2′-aminobenzoylacetate (2-ABA-CoA). The Claisen condensation of anthraniloyl-CoA with malonly-CoA is catalysed by the enzyme PqsD, for which we have previously established simple α-chloroacetamides with a terminal alkyne as chemical probes.^15^ Using competitive labelling, we could identify potent live-cell PqsD inhibitors which were based on the anthranilic acid core of the native substrate (Fig. S1†).

**Fig. 1:**
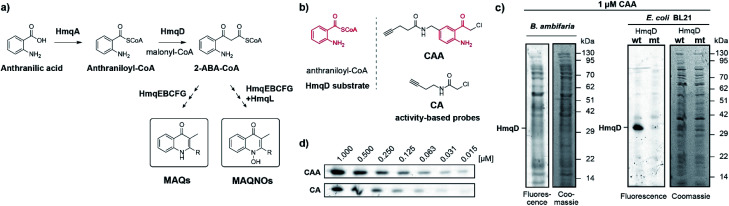
(a) Proposed pathway for the biosynthesis of quinolones by *Burkholderia* species, where HmqD catalyses the Claisen-type condensation reaction of anthraniloyl-CoA with malonyl-CoA to yield 2-ABA-CoA. (b) Probe design and structural analogy of probe CAA with the native HmqD substrate and comparison to the previous probe CA. (c) Fluorescent and Coomassie stained gels of proteomes of live *B*. *ambifaria* and *E*. *coli* overexpressing HmqD labelled with 1 μM of probe CAA. (d) Concentration-dependent labelling of live *E. coli* cells overexpressing HmqD. Wt = wild type HmqD, mt = Cys114Ala mutant HmqD.

We now aimed to synthesize a more specific probe as substrate analogue for HmqD equipped with a terminal alkyne handle for attachment of a fluorescence tag by click chemistry (Fig. 1b). In short, 4-aminobenzylamine was reacted with chloroacetonitrile by Houben–Hoesch acylation to the corresponding α-chloroketone (**2**). Amide coupling with 4-pentynoic acid yielded probe CAA (Scheme 1).

**Scheme 1:**
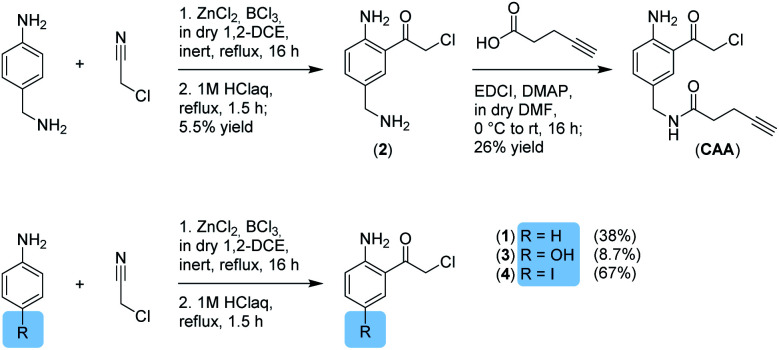
Synthesis of α-chloroketones.

Live cells of *Burkholdera ambifaria* were incubated with probe for 30 min. Unbound probe was removed by washing steps and after cell lysis, a fluorescent tetramethylrhodamine tag was appended *via* bioorthogonal Cu(i) catalysed azide–alkyne cycloaddition. Subsequently, proteins were separated *via* sodium dodecyl sulfate polyacrylamide gel electrophoresis (SDS-PAGE) to allow in-gel fluorescence analysis. Expression levels of HmqD in *Burkholderia ambifaria* were likely too low to result in any fluorescent labelling above background (Fig. 1c).

In accordance, growth phase-resolved proteomic experiments with and without enrichment by pull-down failed to detect any peptides of HmqD suggesting very low abundance of the protein in the native proteome (ESI†). We thus generated an *Escherichia coli* BL21 clone recombinantly expressing HmqD of *B. ambifaria* as model system (Tables S1–S3†). Indeed, labelling of live cells of *E*. *coli* overexpressing HmqD with 1 μM probe CAA resulted in only one major fluorescent band. In contrast, an HmqD mutant with the active site cysteine replaced by alanine (Cys114Ala) was not labelled (Fig. 1c). This indicated not only remarkable selectivity of the probe for HmqD in the background of a live cell proteome but also excellent specificity for the active site nucleophile. Testing the CA and CAA probes side by side in concentration dependence demonstrated that labelling of HmqD in live *E*. *coli* cells was more sensitive with the new CAA probe than with the original CA probe and a fluorescent band was still detectable at only 15 nM of CAA probe (Fig. 1d and S2†). In addition, proteomic experiments with recombinant HmqD expressed in *E*. *coli* identified as site of modification by probe CAA only the active site cysteine C114 out of in total 8 cysteines of HmqD (Fig. S3†). These results validated the CAA probe as tool for competitive live cell profiling of potential HmqD inhibitors (Fig. 2a). We synthesized several α-chloroketones (**1–4**) using the Houben–Hoesch reaction (Scheme 1) and combined them with commercially available compounds (**5–24**) to generate a small library (Fig. 2b). These compounds were screened at 10 μM by pre-incubation with live cells of *E*. *coli* expressing HmqD followed by addition of 1 μM CAA probe, click chemistry and in-gel fluorescence imaging (Fig. 2a). Competitive inhibitors with sufficient cell permeability prevented the labelling by the probe resulting in reduced or complete absence of the HmqD fluorescent band in the background of the live cell proteome. Interestingly, all α-bromoketones were inactive while various α-chloroketones completely inhibited labelling at 10 μM (Fig. 2c and S4†). At higher a concentration of 50 μM, three α-bromoketones inhibited labelling of HmqD *in vitro* but only one showed activity in live cells, suggesting that α-bromoketones may be scavenged by off-targets binding (Fig. S5†).

**Fig. 2:**
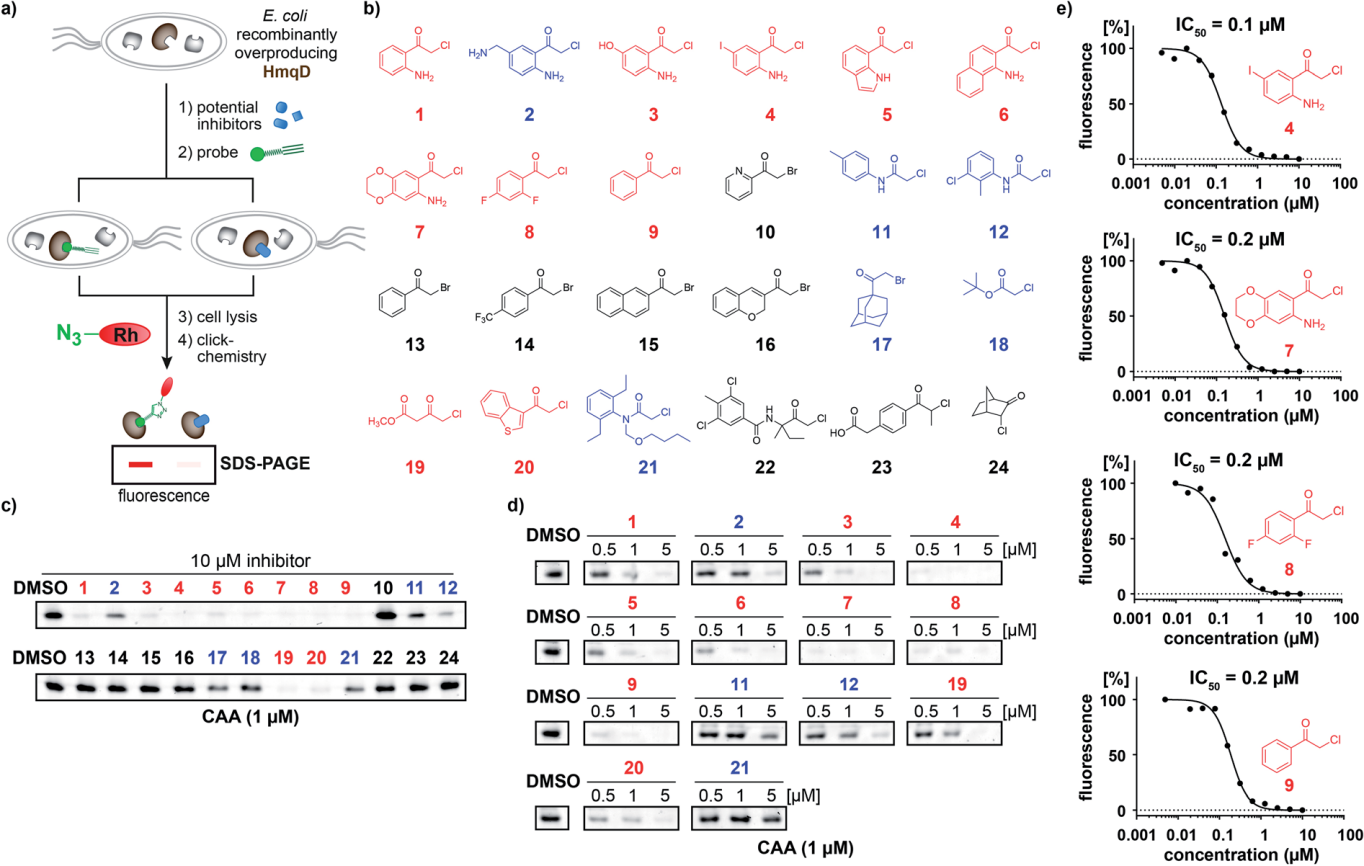
Competitive profiling for HmqD inhibitors. (a) Strategy of live-cell competitive inhibitor discovery using an activity-based probe to screen for active site-directed inhibitors of the target enzyme HmqD. (b) Structures of a focussed compound library featuring electrophilic α-chloroketone, α-chloroamide, and α-bromoketone warheads. (c) Initial competitive live-cell screening of the compound library at 10 μM against HmqD. (d) Dose-down experiments of the preliminary hits identified in (c). (e) Live-cell IC_50_ values for competitive HmqD inhibition. Color code: active compounds with inhibition of competitive labelling ≤1 μM (red), compounds with inhibition ≥5 μM (blue), compounds inactive at 10 μM (black).

The most effective inhibitors were subjected to dose-down experiments with 5 μM, 1 μM and 0.5 μM (Fig. 2d and S6†). Four of them considerably inhibited labelling even at the lowest concentration of 0.5 μM (Fig. 2d). For these compounds a wider range of concentration was used and IC_50_ values were determined by quantification of labelling intensities. The anthranilic acid-derived compounds **7**, **8**, and **9** inhibited labelling with IC_50_ values of 0.2 μM and the most active 5′-iodo-substituted compound **4** even resulted in an IC_50_ of 0.1 μM (Fig. 2e and S7†).

The three most active inhibitors **4**, **7** and **8** were finally tested in cultures of *B. ambifaria* strain AMMD, which has been reported to produce MAQs.^8^ We quantified levels of the six major MAQ derivatives in the culture supernatants using multiple reaction monitoring (MRM) on a triple quadrupole mass spectrometer measuring selective mass transitions of the most intense ions resulting from the fragmentation of precursor ions (Fig. 3a, S8, S9 and Table S4†).^8^ Isolated and purified Δ^2^MHQ was spiked into the growth medium and served as quantification standard to confirm linearity within the detection range (Table S4†). Strikingly, after growing cultures of *B. ambifaria* in presence of inhibitors for 24 h under oxygen limiting conditions, compound **4** and **7** completely blocked MAQ production at concentrations as low as 5 μM (Fig. 3b, S10 and S11†). In contrast, compound **8** only abolished global MAQ production in a higher concentration of 50 μM. Growth was not affected by the most active inhibitor **4** at a concentration of 50 μM over an incubation period of 40 h (Fig. 3c), confirming that low MAQ levels are not artefacts of growth inhibition. Interestingly, under aerobic conditions, MAQ concentrations of *B. ambifaria* peaked in the late exponential growth phase within a narrow time frame of 3 h (Fig. 3c and S12†). The sharp peak and the subsequent complete disappearance of MAQs in culture supernatants indicate a tightly regulated production and suggest the existence of a degradation mechanism. Under oxygen limiting conditions, MAQ levels were considerably lower and continuously produced over a longer period of time in the late stationary growth phase, which implies a modulation of the MAQ biosynthesis by oxygen.

**Fig. 3:**
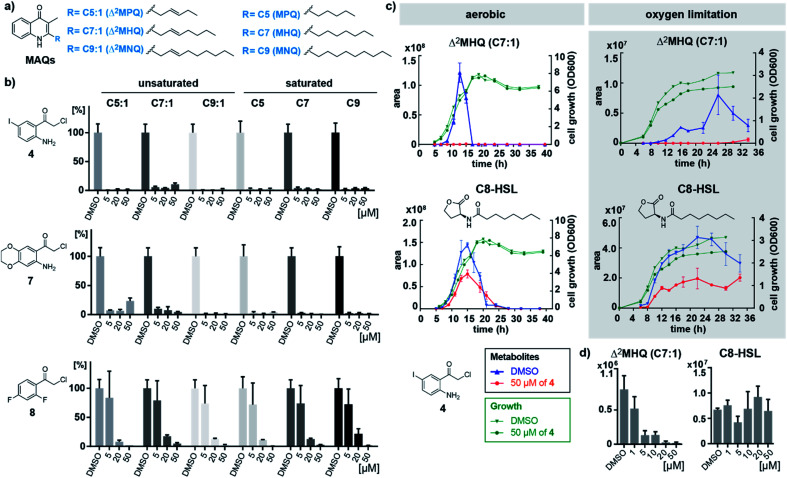
(a) Structures of unsaturated and saturated 3-methyl-2-alkylquinolones (MAQs) with different chain lengths naturally produced by *B*. *ambifaria*. (b) MAQs detected in the culture supernatants of *B*. *ambifaria* treated for 24 h with different concentrations of inhibitors **4**, **7** and **8** under oxygen limiting conditions. Bars represent normalized integrated areas of recorded mass transitions with standard deviation of experiments performed in biological triplicates. (c) Overlay of growth curves and time-resolved mass spectrometric detection of the main quinolone Δ^2^MHQ and the *N*-octanoyl-l-homoserine lactone (C8-HSL) quorum sensing signal of *B*. *ambifaria* grown under aerobic and oxygen limiting culture conditions with 50 μM of inhibitor **4** in comparison to DMSO control. (d) Concentration-dependence of the effect of inhibitor **4** on Δ^2^MHQ and C8-HSL detected in *B*. *ambifaria* cultures after 11 h under aerobic conditions.

To investigate the effect of inhibitors on other quorum sensing systems, we quantified acyl homoserine lactones (AHLs) in the supernatant of *B. ambifaria* AMMD (Table S5†).

Under aerobic conditions highest AHL concentrations were detected between 10 and 20 h of growth, with 3-OH-C10-HSL and C8-HSL as mainly produced AHLs (Fig. S13†). Oxygen limiting conditions resulted in significantly lower AHL concentrations with maximal production in the stationary phase. It is remarkable that production levels as well as timing of quinolone and AHL production appear to be strictly controlled by oxygen levels. Both conditions (normoxic and anoxic) are encountered in the lungs of cystic fibrosis patients.^16^ Consistent with our findings, species of the *Burkholderia cepacia* complex are known to adaptively respond to anaerobic conditions in the cystic fibrosis lung.^17^ While MAQ production was completely inhibited by compound **4**, AHL production was only mildly affected. To confirm this specificity, we profiled MAQ and AHL levels in culture supernatants of *B. ambifaria* grown for 11 h under aerobic conditions treated with a range of concentrations of compound **4**. MAQ production was considerably inhibited already at 1 μM of **4** and further decreased with higher concentrations. In contrast, AHLs showed no concentration-dependent inhibition (Fig. 3d and S15†). These results demonstrate that compound **4** is specific for inhibition of quinolone biosynthesis in *B. ambifaria* AMMD.

To detect potential off-targets of inhibitor **4**, we conducted additional competitive labelling experiments. Proteomic experiments revealed several potential off-targets of the CAA probe (Tables S6 and S7†) and we additionally generated a reactive α-bromoacetamide probe (BA). Even at 10-fold excess, compound **4** did not compete noticeably with the off-target labelling by probe CAA or BA (Fig. S14†), providing further support for a certain level of specificity of inhibitor **4**.

Our results not only show the value of competitive profiling of live cells with activity-based probes, but to the best of our knowledge also provide the first inhibitors of quinolone biosynthesis in the genus *Burkholderia*. Inhibiting quinolone signalling of *Pseudomonas aeruginosa* is an important anti-infective strategy aiming to ameliorate virulence and pathogenicity.^14,18,19^ While in *P*. *aeruginosa* 2-alkylquinolones (HHQ and PQS) are important quorum sensing signals,^20,21^ recent work suggested that MAQs do not induce their own production in *B*. *ambifaria* strain HSJ1 and are probably acting on and regulated *via* the CepI/CepR AHL quorum sensing system.^10^ Although the exact roles of MAQs in the *Burkholderia cepacia* complex still need to be investigated, the homologous AQs of *P*. *aeruginosa* indicate possible roles in suppressing the host immune response, mediating the killing of host cells and modulating interspecies and interkingdom interactions.^22–24^

## Conclusions

In conclusion, we show that a substrate-analogous activity-based probe targeting the central quinolone biosynthesis enzyme HmqD of *B*. *ambifaria* can be applied to find competitive inhibitors in a live cell model and present the first known HmqD inhibitors. The most potent HmqD inhibitors are able to completely block quinolone biosynthesis in cultures of *B*. *ambifaria* even in single-digit micromolar concentrations, while keeping the production of homoserine lactone quorum sensing signals intact. Thus, our compounds represent promising chemical tools for dissecting the role of quinolones in pathogenicity of *Burkholderia* species.

## Author contributions

M. P. and T. B. conceived and designed the study. M. P., V. F., and S. O. synthesized the probes, produced the recombinant proteins, and performed the labelling experiments. M. P. and V. F. extracted and analyzed the metabolites by mass spectrometry. M. P., V. F., S. O., and T. B. analyzed and interpreted the data. M. P. and T. B. wrote the manuscript.

## Conflicts of interest

There are no conflicts to declare.

## Supplementary Material

SC-012-D0SC06167K-s001

SC-012-D0SC06167K-s002
