# Supplementary material for: Inhibiting quinolone biosynthesis of Burkholderia
Source: Chem Sci. 2021 Mar 26;12(20):6908–12. doi: 10.1039/d0sc06167k (PMC8153077; doi:10.1039/d0sc06167k)
Supplement: SC-012-D0SC06167K-s001 [file SC-012-D0SC06167K-s001.pdf]

Electronic Supplementary Material (ESI) for Chemical Science.
This journal is © The Royal Society of Chemistry 2021

Inhibiting quinolone biosynthesis of *Burkholderia*

Michaela Prothiwa, Verena Filz, Sebastian Oehler, Thomas Böttcher

1. Materials

All solvents and chemical reagents for synthesis and LC-MS/MS analysis were purchased from Sigma-Aldrich, VWR or Carl Roth. The LC-MS standards C6-HSL, C8-HSL, C10-HSL, 3-OH-C8-HSL and 3-OH-C10-HSL were obtained from Cayman Chemicals. Flash chromatography was performed using silica gel. NMR spectra were obtained using a Bruker Avance III 400 NMR instrument equipped with a BBFO plus probe and calibrated on the residual solvent peak. Chemical shifts are reported in ppm and coupling constants (*J*) are given in Hz. High-resolution mass spectra were recorded by an ESI-TOF MS (Bruker Daltonics amicroTOFII) equipped with a Chromolith FastGaradient Rp18e 50x2 mm column (Merck). Low-resolution mass analysis was performed using an ESI-IT MS (Bruker Daltonics Esquire 3000plus) with a Nucleoshell 50x2 mm RP-18 2.7 µm column (Macherey-Nagel). Quantitation was performed on a Finnigan™ TSQ® Quantum (Thermo electron corporation) mass spectrometer. For SDS-gel preparation and SDS-PAGE a PeqLab PerfectBlue™ was used. The recording and analysis of the gels was performed with the Fusion-FX7 Advanced of Vilber Lourmat (Eberhardzell, Germany) using the software CaptAdvance. The expression vectors pET-51b(+)/*hmqD* and *E. coli* BL21 pET-51b(+)/*hmqDmutant* (Cys114Ala) were purchased from GeneScript, and the genes were codon optimized for expression in *E. coli*.

2. Biological methods

Preparation of overnight cultures

For preparation of overnight cultures, a small amount of a bacterial cryo-stock (15% glycerol, stored at -80 °C) was inoculated in 5 mL LB-(lysogenic broth)-Lennox medium (LB medium) in sterile 13 mL polypropylene tubes (Sarstedt, ref 62.515.028), supplemented with antibiotics if indicated, and grown for 14-16 h at indicated growth conditions (**Table S1**)

Growth curve analysis of *Burkholderia ambifaria* during inhibitor treatment

In sterile 15 mL polypropylene centrifuge tubes with screw caps (Roth), 4 µL of a 50 mM DMSO Stock of inhibitor **4**, or 4 µL DMSO as vehicle control, was added (0.1 % final DMSO concentration). 4 mL of LB medium and 60 µL of an overnight culture of *Burkholderia ambifaria* AMMD was added per sample. The caps of the sample tubes were slightly opened and fixed in a defined position by tape to ensure equal oxygen delivery to all samples. For oxygen limiting

conditions caps were tightly closed. The samples were incubated until indicated time points at 30 °C (240 rpm). Afterwards, 50 µL LB medium and 50 µL of culture were transferred in a cuvette, mixed and the optical density of cells was measured at a wavelength of λ= 600 nm (OD_{600}) with a photometer of Eppendorf (BioPhotometer® plus). The experiment was performed in 3 biological replicates.

***In situ* labelling of *Burkholderia ambifaria* with probe CAA**

60 µL of an overnight culture of *Burkholderia ambifaria* DSM 16087 was added to 20 mL LB medium in 50 mL sterile polypropylene centrifugal tubes with screw caps (Roth) and grown for 10 h at 37 °C (180 rpm). Then, 2 mL of cell culture was transferred into a 2 mL Eppendorf tube, centrifuged (4500 g, 5 min, 4 °C), and the cells washed with 1 mL PBS. The remaining cell pellet was carefully resuspended in 100 µL PBS before addition of 1 µL of a 0.1 mM CAA probe stock in DMSO. After incubation for 1h at 37 °C in a shaking incubator (180 rpm) the cells were washed two times with 1 mL PBS. For cell lysis, 100 µL PBS was added and the cells lysed by ultrasound treatment (10 % amplitude, 0.5 sec on, 1.0 sec off, 20 pulses, Branson Digital Sonifier), and the cell debris removed by centrifugation (12000 g, 10 min, 4 °C). The resulting lysates were used for click-chemistry and SDS-PAGE.

Competitive labelling of *Burkholderia ambifaria* proteome with probe CAA and BA

60 µL of an overnight culture of *Burkholderia ambifaria* DSM 16087 was added to 15 mL LB medium in 50 mL sterile polypropylene centrifugal tubes with screw caps (Roth) and grown for 10 h at 37 °C (220 rpm). The caps of the tubes were slightly opened and fixed in a defined position to ensure equal oxygen delivery. Then, 2 mL of cell culture was transferred into a 2 mL Eppendorf tube, centrifuged (4500g, 5 min) and the cells were washed with 1 mL PBS. The remaining cell pellet was carefully resuspended in 100 µL PBS before 1 µL inhibitor stock in DMSO was added to obtain final concentrations of 10, 50, 100 and 200 µM. The cell suspension was incubated for 30 min at 37 °C (180 rpm) and lysed using the FastPrep-24 system (MP biomedicals). Cell debris was removed by centrifugation (13500 rpm, 5min, 4 °C) and the supernatant was sterile filtrated. 100 µL of filtrate were transferred to a 1.5 mL Eppendorf tube, 1 µL of 2 mM CAA or BA probe stock in DMSO was added (final concentration 20 µM) and incubated at room temperature for 30 min. Subsequently, click chemistry and SDS-PAGE analysis were performed.

***In situ* labelling of *E. coli HmqD* cells**

E. coli BL21 cells containing pET-51b(+)/hmqD (wild type hmqD) or pET-51b(+)/hmqDmutant (Cys112Ala mutant) were grown in an overnight culture supplemented with 100 µg/mL carbenicillin. On the next day, the culture was inoculated 1:50 in LB media, supplemented with

100 µg/mL carbenicillin and incubated at 37°C (180 rpm) to an OD_{600} of 0.5. Next, 150 µM (final concentration) isopropyl β-D-1-thiogalactopyranoside (IPTG) was added to induce protein expression, and the culture was incubated at 37°C for 30 min (180 rpm). For each sample, 500 µL of induced bacterial culture was transferred into a 1.5 mL Eppendorf tube, the cells pelleted by centrifugation (4500 g, 5 min, 4°C), and the cell pellet washed with 800 µL PBS before re-suspending in 100 µL PBS. 1 µL of inhibitor stock in DMSO or DMSO as vehicle was added for the indicated final concentration, mixed gently and pre-incubated for 15 min at rt. Then, 1 µL of a 100 µM CAA stock in DMSO was added to a final concentration of 1 µM and incubated for 60 min at rt. After incubation, the cells were pelleted by centrifugation (4500 g, 5 min, 4 °C), washed (2 x 800 µL PBS), the cell pellet was re-suspended in 100 µL PBS, lysed by ultrasound treatment (10 % amplitude, 0.5 sec on, 1.0 sec off, 10 pulses, Branson Digital Sonifier), and the cell debris removed by centrifugation (12000 g, 10 min, 4 °C). The resulting lysates were used for click-chemistry and SDS-PAGE. After fluorescence scanning, Coomassie staining was applied to compare protein concentrations in the gel and thereby validate the experiments (Figure S11).

***In situ* determination of IC_{50} values**

E. coli BL21 cells containing pET-51b(+)/hmqD (wild type HmqD) were grown in an overnight culture supplemented with 100 µg/mL carbenicillin. On the next day, the culture was inoculated 1:50 in LB medium, supplemented with 100 µg/mL carbenicillin and incubated at 37°C (180 rpm) to an OD_{600} of 0.5. Next, 150 µM IPTG was added to induce protein expression, and the culture was incubated at 37°C for 120 min (180 rpm). For each sample, 2 mL of induced bacterial culture was transferred in a 2 mL Eppendorf tube, the cells pelleted by centrifugation (4500 g, 5 min, 4 °C), and the cell pellet washed with 1 mL PBS before re-suspending in 100 µL PBS. To this cell suspension, 1 µL of inhibitor stock in DMSO was added at indicated concentrations (n = 3) and incubated for 30 min at 37 °C (180 rpm). Afterwards, the probe CAA was added to give a final concentration of 1 µM and incubated 60 min at rt. The cells were washed (2 x 1 mL PBS), lysed by sonication, cell debris was removed by centrifugation and rhodamine attached via click chemistry. Subsequently, the samples were analyzed by SDS-PAGE and in-gel visualization. Integrated fluorescence intensities of the bands were measured using ImageJ software and IC_{50} values calculated from a dose-response curve generated using GraphPad Prism software.

Click chemistry

Click chemistry was performed using 19.5 µL cell lysate, 1 µL of a 21.5 % SDS solution, 0.5 µL of a 0.325 mM rhodamine azide stock in DMSO, and 1.5 µL of a 2 mM tris((1-benzyl-4-triazolyl)methyl)amine (TBTA) stock in *tert*-Butanol/DMSO (8:2 v/v). To start the cycloaddition

reaction, 1 µL of a freshly prepared 25 mM tris(2-carboxyethyl)phosphine hydrochloride (TCEP) solution in water and 1 µL of a 25 mM $CuSO_4$ stock solution in water was added. The samples were incubated for 1 h at rt and quenched by addition of 2 x SDS loading buffer (63 mM Tris-HCl, 10% (v/v) Glycerol, 2% (w/v) SDS, 0.0025% (w/v) Bromophenolblue, 10% (v/v) β-mercaptoethanol; dissolved in water).

3. Analytical methods

Quantitation of MAQs, MAQNOs and AHLs

LC-MS/MS analysis of MAQs, MAQNOs and AHLs

Ultra-high performance liquid chromatography was performed on a Dionex Ultimate 3000 UHPLC (Thermo Fisher Scientific, Waltham, MA) using a Nucleodur C18 Gravity-SB 100 x 2 mm, 3 µm column (Macherey-Nagel). The flow rate was 0.5 mL/min and the column temperature was set to 40 °C. The injection volume was 5 µL using pick up injection mode. Eluent A was 0.1% formic acid in water and eluent B was acetonitrile. The gradient was 20-100 % B in 10 min, 100 % B for 2 min, 100-20 % B in 1 min and 20 % B for 2 min. MS/MS analysis was performed by a Finnigan™ TSQ® Quantum (Thermo electron corporation) mass spectrometer in the SRM (Selected Reaction Monitoring) scan mode. As ion source a heated-electrospray ionization (HESI-II probe, Thermo scientific) was used. In the optimized conditions the ion spray voltage was 3500 V, vaporizer temperature 300 °C, capillary temperature 380 °C, sheath gas pressure 60 psi, ion sweep gas pressure 2 psi, aux gas 10 psi. The tube lens offset was 80 V and skimmer offset 0. Data was acquired in a mass range of *m/z* 130-350, and MS/MS acquisition of all compounds was performed in positive mode and fixed collision energy of 30 eV. Dwell time was 0.050 sec for all compounds. The software Quan Browser Thermo Xcalibur 3.1.66.10 was used for quantitative analysis. The standard peak area of the product ions was fitted by linear regression versus the known concentrations to generate a standard curve.

Preparation of calibration standards

<u>Δ^2MNQ standard</u>

Isolation of Δ^2MNQ from *Burkhoderia thailandensis:* In total 15x 1 L of LB medium were prepared with 3.5 mL of an *B. thailandensis* overnight culture was inoculated in 1 L LB medium and incubated for 48 h at 37 °C (200 rpm). Afterwards, cultures were centrifuged to pellet cells and the supernatants were extracted 2 x with ethyl acetate (1:1 v/v). The crude extract was purified by flash chromatography (Hex / 10-100% EE gradient) (TELEDYNE ISCO

CombiFlash® Rf; Lincoln, Nebraska, USA), preparative HPLC (H_2O, 50-100% MeOH) (Spot Prep II Liquid Chromatography system; Armen Instrument, France) equipped with a Reprosil 100 C18 250 x 20 mm, 5 µM (Macherey Nagel) and analytical HPLC (H_20, 50-100% MeOH) equipped with an Reprosil 100 C18 250 x 10 mm, 5 µM (Dr. Maisch). The structure of Δ^2MNQ was verified by NMR[1] (**Figure S16**) and HRMS.

^{1}H NMR (600 MHz, MeOD): δ = 8.23 (dd, J = 8.2, 1.5 Hz, 1H, C=OCC**H**), 7.62 (ddd, J = 8.4, 6.8, 1.5 Hz, 1H, NHCCHC**H**), 7.54 (d, J = 8.4 Hz, 1H, NHCC**H**), 7.36 – 7.31 (m, 1H, C=OCCHC**H**), 5.62 – 5.52 (m, 2H, C**H**C**H**), 3.50 (d, J = 4.3 Hz, 2H, CC**H₂**CH), 2.14 (s, 3H, C**H₃**C=O), 2.06 – 1.99 (m, 2H, CHC**H₂**CH₂), 1.38 – 1.30 (m, 2H, CHCH₂C**H₂**), 1.30 – 1.19 (m, 6H, CH₃C**H₂**C**H₂**C**H₂**), 0.84 (t, J = 6.7 Hz, 3H, C**H₃**CH₂); ^{13}C NMR (100 MHz, $CDCl_3$): δ = 179.6, 151.2, 140.6, 135.2, 132.7, 126.2, 125.0, 124.6, 124.5, 118.7, 116.7, 36.4, 33.5, 32.8, 30.3, 29.8, 23.7, 14.4, 10.7; HRMS (ESI-Orbitrap): $C_{19}H_{26}NO$ $[M+H]^+_{calc}$: m/z 284.2014. $[M+H]^+_{found}$: m/z 284.1998 (Δppm: 5.6)

A MeOH stock solution of isolated and purified Δ^2MNQ were prepared at 1 mg/mL in a glass vial and stored at -80 °C up to one month. A series of standard solutions was prepared freshly before LC-MS/MS analysis by serial dilutions of the stock solution in LB medium (standard concentrations and equation see **Table S4**). For each sample, 2.8 mL of standard containing LB medium was transferred in 10 mL headspace vials, 2 mL ethyl acetate was added and vortexed for 5 sec. Then, 1 mL of organic layer was transferred in a clean 1.5 mL glass vial and the solvent evaporated by a gentle stream of nitrogen. Standards were extracted in 3 replicates. For LC-MS/MS analysis, 25 µL acetonitrile/water (1:1) was added to each sample.

AHL standards:

MeOH stock solutions of C6-HSL, C8-HSL, C10-HSL, 3-OH-C8-HSL and 3-OH-C10-HSL were prepared at 0.1 mg/mL in glass vials and stored at -80 °C up to one month. A series of standard solutions was prepared freshly before LC-MS/MS analysis by serial dilutions of the stock solution of each analyte in acetonitrile/water (1:1) (standard concentrations and equations see **Table S5**).

Determination of MAQ levels after inhibition with inhibitors 4, 5 and 7

In general, quinolone levels were determined in 3 replicates. 4 µL of a respective DMSO stock solution of inhibitors or 4 µL DMSO as vehicle control (0.1 % final DMSO concentration) was added in 15 mL polypropylene centrifuge tubes with screw caps (VWR). Afterwards 4 mL LB medium was added in each tube to obtain final inhibitor concentrations of 5, 20 and 50 µM. Then, 60 µL of a *Burkholderia ambifaria* AMMD overnight culture was added. Caps of the

sample tubes were tightly screwed. Samples were incubated for 24 h at 28 °C (240 rpm). Afterwards, samples were centrifuged, supernatants sterile filtered, metabolites extracted and quantified by LC-MS/MS. The experiment was performed in 3 biological replicates.

Quantification of MAQ and AHL levels in dose dependence with inhibitor ***4***

4 µL of a respective DMSO stock solution of inhibitor **4** or DMSO as vehicle control was added in 15 mL polypropylene centrifugal tubes with screw caps (VWR). Then, 4 mL LB medium was added in each tube to obtain final inhibitor concentrations of 1, 5, 10, 20 and 50 µM. Subsequently, 60 µL of a *Burkholderia ambifaria* DSM 16087 overnight culture was added and samples incubated for 11 h at 37 °C (220 rpm). Caps of the tubes were slightly opened and fixed in a defined position to ensure equal oxygen delivery. Afterwards, samples were centrifuged (4500 rpm, 10 min) and supernatants sterile filtrated. 2.8 mL filtrate was transferred to a 10 mL headspace vial, 2 mL ethyl acetate were added and vortexed for 5 seconds. Then, 1 mL of organic layer was transferred to a clean 1.5 mL glass vial and the solvent evaporated under a gentle stream of nitrogen. For LC-MS/MS analysis, 25 µL acetonitrile/water (1:1) was added to each sample. The experiment was performed in 3 biological replicates.

Determination of MAQ, MAQNO and AHL levels during growth with low/high oxygen content

4 µL of a 5 mM inhibitor stock in DMSO or DMSO as vehicle control (0.1 % final DMSO concentration) was added to 4 mL LB medium in 15 mL polypropylene centrifuge tubes with screw caps (VWR). Afterwards 60 µL *B. ambifaria* AMMD overnight culture was added. For aerobic conditions, the caps of the sample tubes were slightly opened and fixed in a defined position by tape to ensure equal oxygen delivery to all samples. For oxygen limiting conditions caps were tightly closed. The samples were incubated for indicated time points at 30 °C (240 rpm). Afterwards, samples were centrifuged, supernatants sterile filtered, metabolites extracted and quantified by LC-MS/MS. The experiment was performed in 3 biological replicates.

Extraction of MAQs and AHLs from supernatant

2.8 mL of sterile supernatant was transferred in 10 mL headspace vials (VWR, VWRI548-0237), 2 mL ethyl acetate was added, closed with a magnetic screw cap (silicone/PTFE, LABSOLUTE, WC/07621126) and vortexed thoroughly for 5 sec. Then, 1 mL of the organic layer was transferred in a clean 1.5 mL glass vial (in case of poor layer separation vials were centrifuged at low speed). The solvent evaporated by a gentle stream of nitrogen. The extract was stored at -80 °C until measurement. For analysis, 25 µL acetonitrile/water (1:1) was added to each sample.

Proteomic LC-MS/MS analysis

Proteomic analysis of HmqD after in situ labelling in E. coli cells with probe CAA

For proteomic analysis, *in situ* labelling of *E. coli* HmqD cells was performed as described above, and 2 x SDS loading buffer was added directly to the resulting lysates. After SDS-PAGE and Coomassie staining, the corresponding protein band was cut out of the gel. Tryptic digestion, sample preparation and LC-MS/MS analysis were performed by the Proteomics facility of the University of Konstanz: Coomassie gel bands were washed in MilliQ for 15 sec, bands cut as close as possible and placed in Eppendorf tubes. For reduction and alkylation, a solution of 10 mM DL-dithiothreitol (DTT) in 50 mM $(NH_4)_2CO_3$ was added and incubated for 60 min at 56 °C. The DTT solution was removed and a solution of 50 mM iodoacetamide in 50 mM $(NH_4)_2CO_3$ was added and incubated for 60 min at rt. In a final washing step, the gel pieces were washed with 100 µL MilliQ water and 100 µL 50 mM $(NH_4)_2CO_3$ solution, then the solution was removed, and the gel dehydrated with acetonitrile/MilliQ water (3:2). The washing step was repeated until the gel pieces turned colorless. Next, the dehydration solution was removed, and acetonitrile was added for 10 sec before drying on air until complete dryness. The gel pieces were incubated for 45 sec on ice in a freshly prepared cold buffer containing trypsin (10.0 ng/µL) and 50 mM $(NH_4)_2CO_3$. Afterwards the solution was removed, and the gel incubated overnight in 50 mM $(NH_4)_2CO_3$ solution at 37 °C. On the next day the supernatant was removed, and peptides were extracted by incubation in acetonitrile / 0.1 % TFA in MilliQ water (3:2) for 60 sec at rt. The elution was collected, and the gel incubated in acetonitrile for 15 sec at rt. The combined washes and elutions were dried via SpeedVac and analyzed by an Orbitrap Fusion with EASY-nLC1200 instrument.

Proteomic analysis of B. ambifaria AMMD without pulldown

500 µL of a *B. ambifaria* AMMD overnight culture was added to 50 mL LB medium in a glass culture flask capped with aluminum foil and incubated at 37 °C, 180 rpm for 6h, 8h and 10h. At each time point labelling with probe CAA was performed in triplicates: 3 mL culture was transferred into a 15 mL polypropylene flask, cells were pelleted at low speed and the supernatant removed. Cells were re-suspended in 100 µL PBS and 1 µL of a 100 µM CAA stock in DMSO was added, the suspension carefully shaken and incubated at rt for 1h. Afterwards the cells were washed with PBS and the cell pellet frozen at -20 °C. For cell lysis, cells were thawed up, resuspended in 300 µL PBS and lysed by bead disruption (FastPrep-24^{TM} 5G beat beating grinder and lysis system). After pelleting cell debris, the supernatant was sterile filtered and frozen at -80 °C until proteomic analysis. For proteomic analysis, proteins

in the supernatant were digested by Trypsin and analyzed by LC-MS/MS (performed by the Proteomics facility of the University of Konstanz as described above). Results for detected peptides modified with probe CAA at each time point are shown in an additional supporting excel sheet.

Proteomic analysis of B. ambifaria AMMD <u>*with pulldown*</u>

For each sample, 16 mL of a *B. ambifaria* AMMD culture was grown in LB medium at 30 °C (220 rpm) in PP culture flasks. After 6h or 10h cells were pelleted at low speed and the supernatant removed. Cells were re-suspended in 100 µL PBS and 1 µL of a 10 mM CAA stock in DMSO was added, the suspension carefully shaken and incubated at 37 °C for 30 min. Cells were washed with PBS and for cell lysis resuspended in 500 µL PBS before lysing via bead disruption (FastPrep). After pelleting cell debris, the supernatant was sterile filtered. Per sample, 250 µL of a 1 mg/mL proteome was incubated with 1.5 µL of a 10 mM azide-containing biotin linker, 5 µL of a 52 mM TCEP stock, 15 µL of a 2 mM TBTA stock in *tert*-butanol/DMSO (8:2 v/v) and 5 µL of a 50 mM $CuSO_4$ stock for 1 h. Afterwards proteins were precipitated by adding 1 mL cold acetone (-80 °C). The mixture was vortexed and stored over night at -80 °C in a glass vial. Precipitated proteins were pelleted for 15 min at 16900 x g (4 °C), the supernatant discarded, and the protein pellet washed with -80 °C cold methanol (2x, resuspended by sonication). Then, the protein pellet was resuspended in 500 µL 0.4% SDS in PBS at rt by sonication. For avidin-bead enrichment, 50 µL bead suspension (Pierce™ Avidin Agarose, Thermo Scientific™, cat.no. 20219) was transferred in a low-protein binding eppendorf tube and washed with 0.4% SDS in PBS (3x) (centrifugation at 400 x g, 3 min, rt). Protein samples were centrifuged (18000 x g, 5 min, rt), 500 µL protein solution was transferred in avidin-bead containing eppendorf tubes and incubated for 1h under careful shaking. Afterwards, beads were washed with 1 mL 0.4% SDS in PBS (3x, 400 x g, rt), 1 mL 6M urea in milliQ water (2x, 400 x g, rt) and 1 mL PBS (3x, 400 x g, rt). For proteomic analysis, proteins on beads were digested by trypsin and analyzed by LC-MS/MS (performed by the Proteomics facility of the University of Konstanz as described above).

4. Synthesis and characterization

Standard procedure to synthesize chloromethyl ketones

In accordance to the literature,[2] $ZnCl_2$ (4.0 eq.) was dried under high vacuum and under nitrogen atmosphere dry 1,2-dichloroethane was added. Aniline (1.0 eq.) and chloroacetonitrile (3.4 eq.) were added and BCl_3 (1 M in dichloromethane, 3.0 eq.) was added slowly. The mixture was heated to reflux (90°C, oil bath) for 15 h. It was cooled to room temperature, 1 M aq. HCl was added and heated to reflux (100°C, oil bath) for 90 min. After cooling to room temperature, DCM and water were added and the aqueous layer was extracted with DCM (3x). The organic layers were combined, dried over $MgSO_4$ and the solvent evaporated.

1-(2-Aminophenyl)-2-chloroethan-1-one (1)

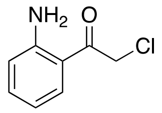

Synthesis according to the standard procedure to synthesize chloromethyl ketones. Scale: $ZnCl_2$ (4.4 g, 32 mmol, 4.0 eq.), dry 1,2-dichloroethane (24.0 mL), aniline (0.75 mL, 8 mmol, 1.0 eq.), chloroacetonitrile (1.75 mL, 28 mmol, 3.4 eq.), 1 M BCl_3 in dichloromethane (24 mL, 3.0 eq.) and in the second step 1 M aq. HCl (24 mL). After cooling to room temperature, DCM (200 mL) and water (200 mL) were added and the aqueous layer was extracted with DCM (3 x 100 mL). The organic layers were combined, dried over $MgSO_4$ and evaporated to give a brown solid (745 mg). Flash column chromatography on silica gel was performed in petroleum ether/ethyl acetate (PE/EA) 7:1 to yield pure product as yellow solid (235 mg, 1.4 mmol, 17%). ^{1}H NMR (400 MHz, C_6D_6): δ = 7.07-7.05 (m, 1H, O=CCC**H**), 6.94-6.90 (m, 1H, NH_2CCHC**H**), 6.33-6.29 (m, 1H, NH_2CCHCHC**H**), 6.07-6.05 (m, 1H, NH_2CC**H**), 5.84 (s, 2H, N**H$_2$**), 3.94 (s, 2H, C**H$_2$**Cl); ^{13}C NMR (101 MHz, C_6D_6): δ = 192.5, 151.5, 134.8, 131.8, 117.4, 115.5, 115.4, 46.2; R_f: 0.62 (PE/EA 5:1). HRMS (ESI-TOF): C_8H_9ClNO $[M+H]^+_{calc}$: m/z 170.0376. $[M+H]^+_{found}$: m/z 170.0364 (Δppm: 1.8).

1-(2-Amino-5-(aminomethyl)phenyl)-2-chloroethan-1-one (2)

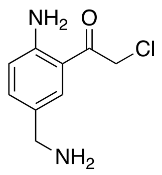

Synthesis according to the standard procedure to synthesize chloromethyl ketones. Scale: $ZnCl_2$ (4.46 g, 32.7 mmol, 4 eq), 51 mL dry 1,2-dichloroethan, 4-aminobenzylamine (927 µL, 8.19 mmol, 1 eq), chloroacetonitrile (1.78 mL, 28.2 mmol, 3.44 eq), 1 M BCl_3 in dichloromethane (24.6 mL, 24.6 mmol, 3 eq) and in the second step 51 mL 1 M HCl_{aq}. The suspension was diluted with 50 mL DCM and 50 mL water. The organic phase was separated to neutralise the aqueous phase with Na_2CO_3 to pH 8. The aqueous phase was diluted with 100 mL water and 200 mL DCM and the suspension filtered. The filter cake was washed with water (100 mL), DCM (250 mL) and EE (250 mL) and the filtered aqueous phase extracted with DCM (5 x 100 mL). The organic phases were combined and dried over $MgSO_4$. After solvent evaporation, the crude was taken up in 10 mL cold chloroform by sonification to discard the formed precipitate and evaporate the solvent of the filtrate. This step was repeated three times to receive the product as yellow solid (90 mg, 5.5% yield).
^1H NMR (400 MHz, $CDCl_3$): δ = 7.57-7.54 (d, J = 1.8 Hz, 1H, O=CCC**H**), 7.31-7.27 (dd, J = 8.5, 1.8 Hz, 1H, NH_2CCHC**H**), 6.70-6.66 (d, J = 8.5 Hz, 1H, NH_2CC**H**), 6.27 (br.s, 2H, N**H_2**), 4.70 (s, 2H, C**H_2**Cl), 3.78 (s, 2H, C**H_2**NH_2), 1.46 (br.s, 2H, N**H_2**); ^{13}C NMR (101 MHz, $CDCl_3$): δ = 192.5, 150.3, 135.1, 130.8, 129.0, 118.1, 115.1, 46.8, 45.9; R_f = 0.13 (DCM/MeOH 9:1, 1% diethylamine); HRMS (ESI-TOF): $C_9H_{12}ClN_2O$ $[M+H]^+_{calc}$: m/z 182.0367. $[M+H]^+_{found}$: m/z 182.0364 (Δppm: 1.8).

1-(2-Amino-5-hydroxyphenyl)-2-chloroethan-1-one (3)

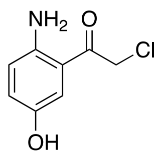

Synthesis according to the standard procedure to synthesize chloromethyl ketones. Scale: $ZnCl_2$ (2.50 g, 18.33 mmol, 4 eq), 28 mL dry 1,2-dichloroethan, 4-aminophenol (0.50 g, 4.58 mmol, 1 eq), chloroacetonitrile (997 µL, 15.8 mmol, 3.44 eq), 1 M BCl_3 in dichloromethane (13.7 mL, 13.7 mmol, 3 eq) and in the second step 28 mL 1 M HCl_{aq}. Reaction mixture was diluted with 50 mL DCM, 50 mL H_2O and the aqueous phase extracted with DCM (3 x 50 mL), the pH of the aqueous phase adjusted to 5 with 8 M $NaOH_{aq}$ (to increase

yield use Na_2CO_3) and again extracted with EE (4 x 50 mL). The combined organic phases were dried over $MgSO_4$, the solvent evaporated, and the crude purified *via* column chromatography (PE:EE, 1:1). The product was obtained as orange-brown solid (74 mg, 8.7% yield).
^{1}H NMR (400 MHz, DMSO-d_6): δ = 8.75 (s, 1H, O**H**), 7.03-7.01 (d, J = 2.8 Hz, 1H, OHCC**H**C), 6.91-6.87 (dd, J = 8.9, 2.8 Hz, 1H, NH_2CCHC**H**), 6.77 (br.s, 2H, N**H₂**), 6.71-6.67 (d, J = 8.9 Hz, 1H, NH_2CC**H**), 4.92 (s, 2H, C**H₂**Cl); ^{13}C NMR (100 MHz, DMSO-d_6) δ = 191.9, 146.4, 145.0, 125.0, 118.5, 114.4, 114.3, 47.6; R_f = 0.23 (PE/EE); HRMS (ESI-TOF): $C_8H_9ClNO_2$ $[M+H]^+_{calc}$: m/z 186.0316. $[M+H]^+_{found}$: m/z 186.0311 (Δppm: 2.7).

1-(2-Amino-5-iodophenyl)-2-chloroethan-1-one (4)

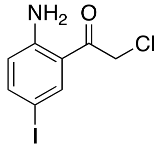

Synthesis according to the standard procedure to synthesize chloromethyl ketones. Scale: $ZnCl_2$ (4.98 g, 36.5 mmol, 4 eq), 57 mL dry 1,2-dichloroethan, 4-Iodaniline (2.00 g, 9.13 mmol, 1 eq), chloroacetonitrile (1.98 mL, 31.4 mmol, 3.44 eq), 1 M BCl_3 in dichloromethane (27.4 mL, 27.4 mmol, 3 eq), and in the second step 57 mL 1M aqueous HCl. After letting the mixture cool to rt, the reaction mixture was diluted with 100 mL DCM and 100 mL water to extract the aqueous phase with DCM (3 x 100 mL). The combined organic phases were dried over $MgSO_4$, the solvent was evaporated, and the crude product purified by column chromatography with a polarity gradient (PE/EE, 12:1 → 8:1 → 4:1) to yield the product as yellow solid (1.81 g, 67% yield).
^{1}H NMR (400 MHz, $CDCl_3$): δ = 7.89-7.87 (d, J = 1.9 Hz, 1H, ICC**H**), 7.52-7.48 (dd, J = 8.8, 1.9 Hz, 1H, NH_2CCHC**H**), 6.51-6.48 (d, J = 8.8 Hz, 1H, NH_2CC**H**), 6.35 (br.s, 2H, N**H₂**), 4.63 (s, 2H, C**H₂**Cl); ^{13}C NMR (100 MHz, $CDCl_3$): δ = 191.6, 150.5, 143.4, 139.0, 119.9, 117.5, 75.5, 46.5; R_f = 0.27 (PE/EE, 8:1); HRMS (ESI-TOF): C_8H_8ClINO $[M+H]^+_{calc}$: m/z 295.9334. $[M+H]^+_{found}$: m/z 295.9328 (Δppm: 1.9).

***N*-(4-Amino-3-(2-chloroacetyl)benzyl)pent-4-ynamide (CAA)**

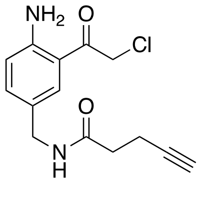

4-Pentynoic acid (3.52 mg, 0.026, 1 eq) was dissolved in 1 mL dry DMF under inert atmosphere and cooled with an ice bath to 0 °C. The acid was pre-activated by the addition of N-(3-dimethylaminopropyl)-N′-ethylcarbodiimide hydrochloride (7.15 mg, 0.037 mmol, 1.04 eq) and DMAP (3.5 mg, 0.029 mmol, 0.8 eq) for 5 min. **Compound 2** (10.0 mg, 0.050 mmol, 1.4 eq) was added in one portion and the mixture was stirred at 0 °C for 10 min and then at rt overnight. The solvent was evaporated, and the residue taken up in DCM (10 mL) and water (5 mL) by sonification. The aqueous phase was extracted with DCM (5 x 7 mL) and the combined organic phases were washed with water (2 x 7 mL) and brine (7 mL). The organic phase was dried over $MgSO_4$ and the solvent evaporated. The crude was purified *via* preparative HPLC (apparatus from Shimadzu equipped with a VP Nucleodur® 100-5 silica column (250 x 10 mm, *Macherey-Nagel*) connected to a SPD-M20A photodiode array detector running at a flow rate of 2.5 mL/min with following gradient: 0-5 min 100% B, 5-15 min 100-95% B, 15-25 min 95% B, 25-30 min 95-90% B, 30-40 min 90% B, 40-45 min 90-100% B, 45-60 min 100% B; solvent A: MeOH, solvent B: DCM) to yield the product as yellow solid (2.6 mg, 26% yield).
^{1}H NMR (400 MHz, $CDCl_3$): δ = 7.55-7.53 (m, 1H, C**H**CC=O), 7.40-7.28 (m, 1H, CH_2CC**H**CH, obscured by $CHCl_3$), 6.68-6.65 (d, J = 8.5 Hz, 1H, CH_2CCHC**H**), 5.88 (br.s, 2H, N**H₂**), 4.67 (s, 2H, C**H₂**Cl), 4.37-4.33 (d, J = 5.6 Hz, 2H, NHC**H₂**), 2.60-2.54 (td, J = 6.8, 2.5 Hz, 2H, HC≡C**H₂**), 2.45-2.41 (t, J = 6.8 Hz, 2H, HC≡CCH_2C**H₂**), 2.00-1.98 (t, J = 2.5 Hz, 1H, **H**C≡C); ^{13}C NMR (100 MHz, $CDCl_3$): δ = 192.5, 171.0, 150.7, 135.4, 130.3, 125.8, 118.2, 115.0, 83.1, 69.6, 46.6, 43.2, 35.5, 15.1; R_f = 0.58 (DCM/MeOH, 9:1); HRMS (ESI-TOF): $C_{14}H_{16}ClN_2O_2$ $[M+H]^+_{calc}$: m/z 279.0895. $[M+H]^+_{found}$: m/z 279.0891 (Δppm: 1.3).

2-Bromo-*N*-(but-3-yn-1-yl)acetamide (BA)

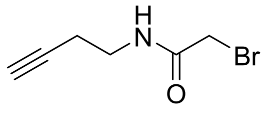

To a solution of 1-amino-3-butyne (1 mmol, 82 µL, 1 eq) in dry DCM (2.5 mL) was added trimethylamine (1.1 mmol, 153 µL, 1.1 eq) under nitrogen atmosphere. Under stirring, the solution was cooled to 0 °C and bromoacetylchloride (1.2 mmol, 99 µL, 1.2 eq) was added. The solution was stirred for 60 min at 0 °C. The reaction mixture was allowed to warm to room temperature and stirred for additional two hours. Afterwards, water (5 mL) was added and extracted with DCM (3 x 5 mL). The combined organic layers were dried over anhydrous $MgSO_4$ and concentrated to yield BA as a colorless oil (169.7 mg, 89.3 %).

^{1}H NMR (400 MHz, $CDCl_3$): δ = 6.84 (s, 1H, N**H**), 3.89 (s, 2H, C**H**$_2$Br), 3.54 – 3.43 (m, 2H, HC≡CCH$_2$C**H$_2$**), 2.46 (td, J = 6.4, 2.5 Hz, 2H, HC≡CC**H$_2$**), 2.07 (t, J = 2.6 Hz, 1 H, **H**C≡C); ^{13}C NMR (101 MHz, $CDCl_3$) δ 165.47, 80.86, 70.44, 38.62, 29.10, 19.18; R_f = 0.33 (PE/EE 6:4); HRMS (ESI-TOF): C_6H_9BrNO $[M+H]^+_{calc}$: m/z 189.9867. $[M+H]^+_{found}$: m/z 189.9865 (Δppm: 1.2).

5. Supporting figures and tables

Table S1. Bacterial strains.

Strain	Growth conditions
Burkholderia ambifaria AMMD (DSM 16087)	LB medium, 28-37 °C, 240 rpm
Burkholderia thailandensis (DSM13276)	LB medium, 37 °C, 200 rpm
E. coli BL21 pET-51b(+)/*hmqD*	LB medium, 37 °C, 180 rpm
E. coli BL21 pET-51b(+)/*hmqDmutant* (Cys114Ala)	LB medium, 37 °C, 180 rpm

Table S2. Gene constructs.

Construct	Description
pET-51b(+)/*hmqD*	Expression vector containing a gene insert coding for the protein HmqD of *B. ambifaria* AMMD with an N-terminal Strep-tag II (purchased from GeneScript) The 5' ATG codon was removed before codon optimization for expression in *E. coli*.
pET-51b(+)/*hmqDmutant* (Cys114Ala)	Expression vector containing a gene insert coding for the mutant protein HmqD Cys114Ala of *B. ambifaria* AMMD with an N-terminal Strep-tag II (purchased from GeneScript) The 5' ATG codon was removed before codon optimization for expression in *E. coli.*

Table S3. Protein sequences

Protein	Sequence
HmqD (*B. ambifaria* AMMD)	MTARLVIAGMGHALPDRIVSNDEVAGMIDTSDAFIRERTGVVARRYLAPDQHLADLAC<br>PAAERAMADAGVTARDVDLLIVNTLSPDHHDPSQACYIQPRLGLREIPCFDIRAQ**C**SG<br>GLYGIEIARHFLASGLYRNVLLICAEALSRRIDSSNAGRNLSILLSDGAAALLLQATDHP<br>AHGLIDLTLGADGTQFDLLSTEAPGARRPRFIDADDIAAGRHHFRMKGKPMFEDATR<br>RIVDACRQMLDKHRLTMSDIGLVVPHQPNLRILDAVIGQLGLPRERCMISVDQLGNMA<br>SAAFPVALAIARELGRMPAGQLNLFVTYGAGATWACALYRS
HmqDm (Cys114Ala mutant; *B. ambifaria* AMMD)	MTARLVIAGMGHALPDRIVSNDEVAGMIDTSDAFIRERTGVVARRYLAPDQHLADLAC<br>PAAERAMADAGVTARDVDLLIVNTLSPDHHDPSQACYIQPRLGLREIPCFDIRAQ**A**SG<br>GLYGIEIARHFLASGLYRNVLLICAEALSRRIDSSNAGRNLSILLSDGAAALLLQATDHP<br>AHGLIDLTLGADGTQFDLLSTEAPGARRPRFIDADDIAAGRHHFRMKGKPMFEDATR<br>RIVDACRQMLDKHRLTMSDIGLVVPHQPNLRILDAVIGQLGLPRERCMISVDQLGNMA<br>SAAFPVALAIARELGRMPAGQLNLFVTYGAGATWACALYRS

(native protein sequences (without N-terminal Strep-Tag II); active site residue is marked)

Supporting Table S4. SRM transitions, standard equations and concentrations of analyzed MAQs.

Retention time [min]	Substance	Transition [*m/z*]	Standard equation, R-value	Standard concentrations [ng/mL]
4.75	**Δ^2MPQ (C5:1)**	228/173		
5.14	**MPQ (C5)**	230/173		
6.26	**Δ^2MHQ (C7:1)**	256/173		
6.56	**MHQ (C7)**	258/173		
7.58	**Δ^2MNQ (C9:1)**	284/173	log(Y) = 6.07599+0.988976*log(X) R^2 = 0.9983	0.04, 0.4, 4, 40, 400
7.98	**MNQ (C9)**	286/173		

Table S5. Retention time, SRM transitions, standard equations and concentrations of analyzed AHLs.

Retention time [min]	Substance	Transition [*m/z*]	Standard equation, R-value	Standard concentrations [µg/mL]
3.09	**C6-HSL**	200/102	Y = -970091+1.0173e +006*X-4243.93*X^2 R^2 = 0.9962	100, 50, 25, 12.5, 6.25, 3.13, 1.56
3.43	**3-OH-C8-HSL**	244/102	Y = -524224+4.60951e +006*X-19473.2*X^2 R^2 = 0.9998	100, 50, 25, 12.5, 6.25, 3.13, 1.56, 0.78
5.49	**3-OH-C10-HSL**	272/102	Y = 24877.5+5.48939e +006*X-21466.5*X^2 R^2 = 0.9998	100, 50, 25, 12.5, 6.25, 3.13, 1.56, 0.78
5.50	**C8-HSL**	228/102	Y = -510969+4.78321e +006*X-15006.2*X^2 R^2 = 0.9999	100, 50, 25, 6.25, 3.13, 1.56
7.12	**C10-HSL**	256/102	Y = 535493+2.10143e +006*X-8276.99*X^2 R^2 = 0.9998	100, 50, 25, 12.5, 6.25, 3.13, 1.56, 0.78

Table S6: Assigned off-target proteins according to peptides modified with probe CAA in the proteome of *B. ambifaria* after 6, 8 and 10 h incubation at 37 °C. (protein accession number HmqD: Q0B3G0)

Protein	Peptide	Protein Accession Number	# PSMs		
			6 h	8 h	10 h
5-methyltetrahydropteroyltriglutamate-homocysteine methyltransferase	1	Q0B6M7	28	31	18
	2		7	10	6
	3		1	1	6
	4		1	2	
Alkyl hydroperoxide reductase C	1	Q0BED9	24	24	36
	2		10	8	9
	3		5	2	4
Glyceraldehyde-3-phosphate dehydrogenase	1	Q0BIC5	12	12	20
Succinate--CoA ligase [ADP-forming] subunit alpha	1	Q0BC72	12	13	9
	2		3	4	4
Thioredoxin reductase	1	Q0BHI0	11	4	
	2		3	1	
Citrate synthase	1	Q0BAF2	7	4	1
	2		4	3	1
	3			1	
	4			1	
Thioredoxin	1	Q0BEV1	7	5	14
S-adenosylmethionine synthase	1	Q0BAY8	6	6	6
	2		3		1
Fructose-1,6-bisphosphate aldolase	1	Q0BC94	6	6	4
	2				4
Succinate semialdehyde dehydrogenase	1	Q0B6G1	6	3	5
Elongation factor G	1	Q0BJ49	6		2

Table S7: Assigned off-target proteins according to peptides after affinity enrichment (pull-down) with probe CAA in the proteome of *B. ambifaria* after 6 and 13 h incubation at 37 °C. (protein group accession HmqD Q0B3G0)

Protein	Protein Group Accessions	# PSMs	
		6 h	13 h
Elongation factor Tu	Q0BJ48		13
3-Methylcrotonoyl-CoA carboxylase, alpha subunit	Q0B6T8		12
Thiol-disulfide interchange protein	Q0BBE7	3	10
5-Methyltetrahydropteroyltriglutamate-homocysteine methyltransferase	Q0B6M7		9
50S Ribosomal protein L7/L12	Q0BJ55		7
10 kDa Chaperonin	Q0BHT2		7
Phenylacetic acid degradation protein paaN	Q0BIL6		7
Uncharacterized protein probably involved in high-affinity Fe2+ transport	Q0BDH0		6
30S Ribosomal protein S20	Q0BCG7		6
30S Ribosomal protein S4	Q0BJ21		5
50S Ribosomal protein L2	Q0BJ43		5
50S Ribosomal protein L23	Q0BJ44		5
60 kDa Chaperonin 1	Q0BHT1		5
50S Ribosomal protein L6	Q0BJ31		5
30S Ribosomal protein S10	Q0BJ47		5
Elongation factor G	Q0BJ49		5
50S Ribosomal protein L31 type B	Q0BEU6	2	5
Acetyl-CoA acetyltransferase	Q0B2W0		5
50S Ribosomal protein L29	Q0BJ38		5
Citrate synthase	Q0BAF2		5
Thioredoxin	Q0BEV1	2	4
Acyl carrier protein	Q0BH14		4
30S Ribosomal protein S15	Q0BDC5		4
Peptidase M48, Ste24p	Q0BBW0		4
50S Ribosomal protein L15	Q0BJ27		4
Biotin carboxyl carrier protein of acetyl-CoA carboxylase	Q0BIF7	3	4
50S Ribosomal protein L24	Q0BJ35	2	4
Cold-shock DNA-binding protein family	Q0B8R9		4
50S Ribosomal protein L16	Q0BJ39		4
50S Ribosomal protein L18	Q0BJ30		4
3-Hydroxyacyl-[acyl-carrier-protein] dehydratase FabZ	Q0BE26		4
Uncharacterized protein	Q0BAG1		4

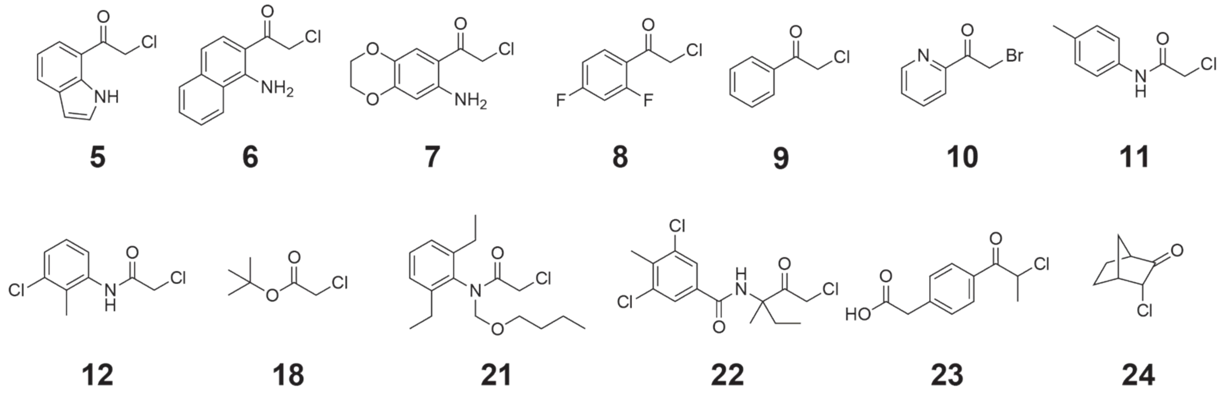

Supporting Figure S1: Live cell inhibitors of PqsD used in this study.[3]

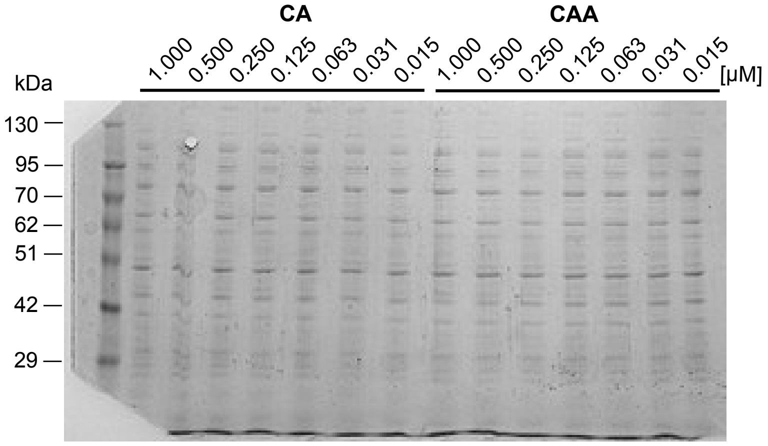

Supporting Figure S2: Coomassie staining of SDS-gel of inhibitor screening experiments shown in **Figure 1d**.

MS of peptide AQC*SGGLYGIEIAR (Cys*114) labeled by probe CAA:

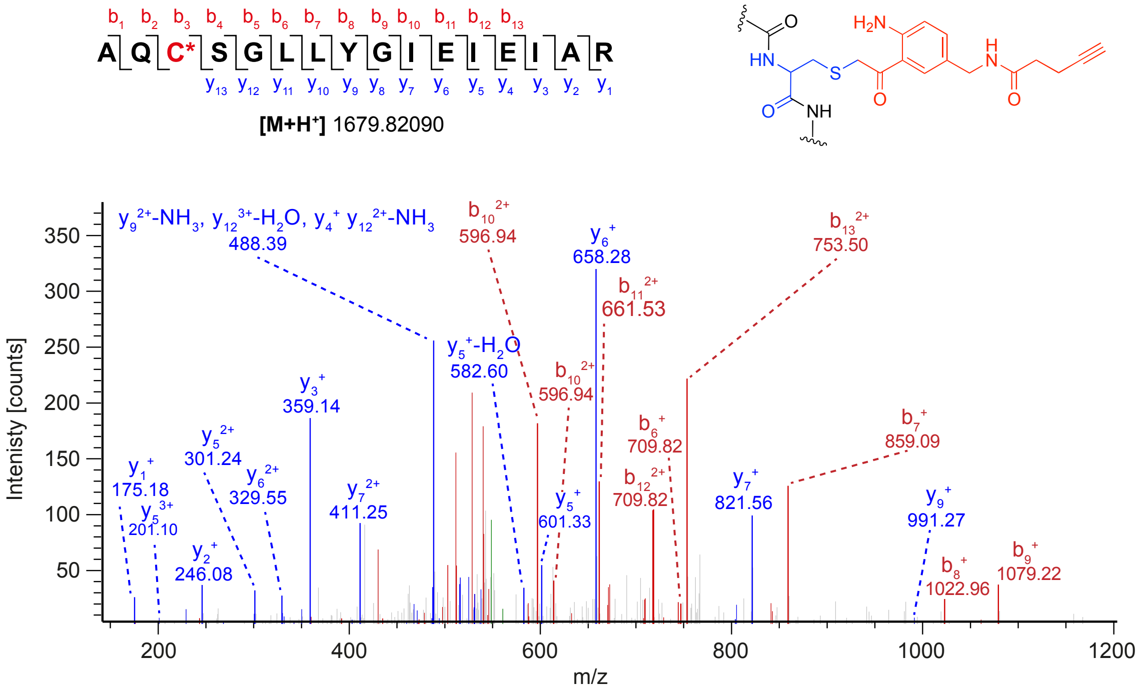

Supporting Figure S3: Tryptic peptide fragment covalently modified by probe CAA after *in situ* labelling of *E. coli* BL21 cells overexpressing HmqD. In the background of whole *E. coli* cell lysate only peptide fragments with CAA bound to the active site cysteine of HmqD were detected.

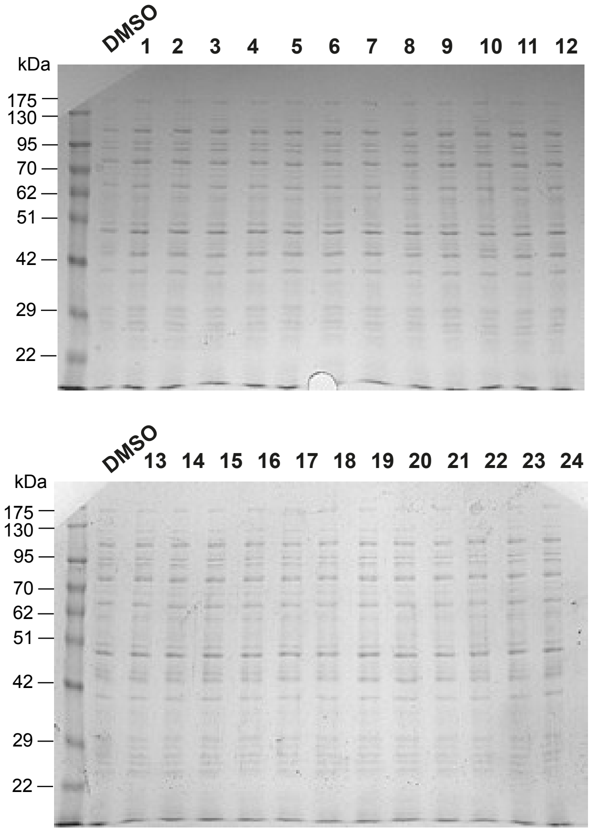

Supporting Figure S4: Coomassie staining of SDS-gels of inhibitor screening experiments shown in **Figure 2c**.

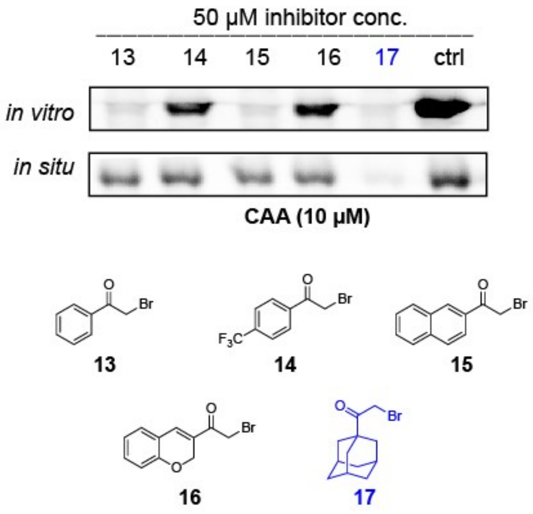

Supporting Figure S5: Competitive labelling of recombinant HmqD (*in vitro*) and *E. coli* cells recombinantly producing HmqD (*in situ*).

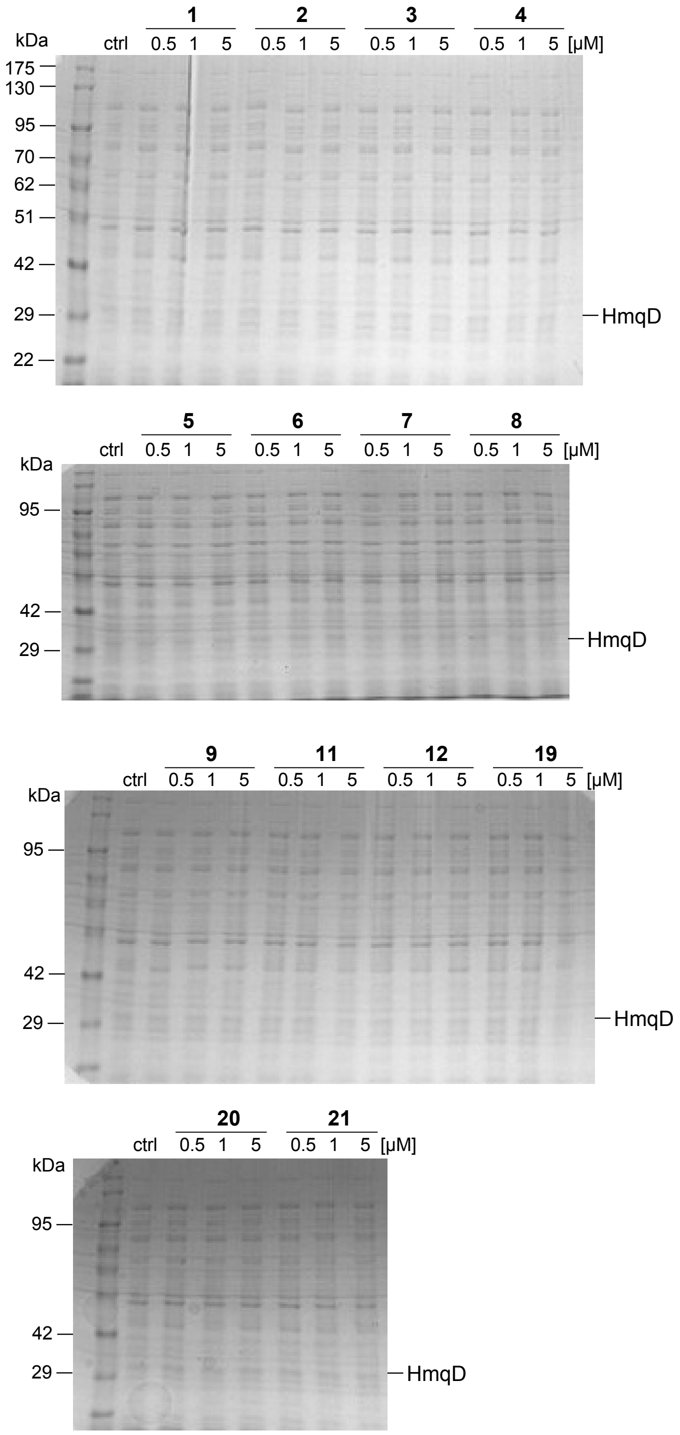

Supporting Figure S6: Coomassie staining of SDS-gels of dose-down experiments shown in **Figure 3d**.

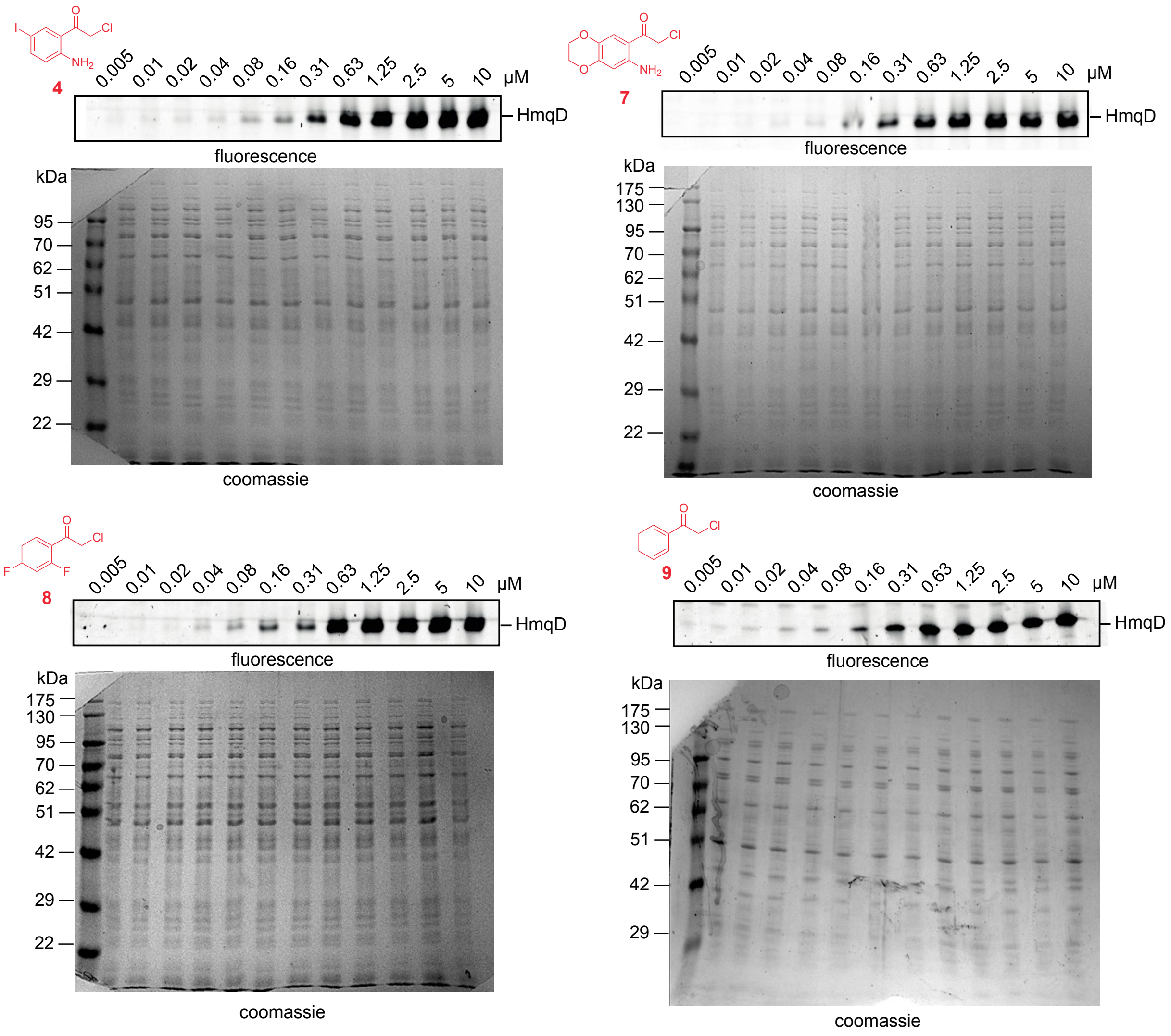

Supporting Figure S7: Fluorescence images and Coomassie staining of SDS-gels of IC_{50} value determination shown in **Figure 3e**.

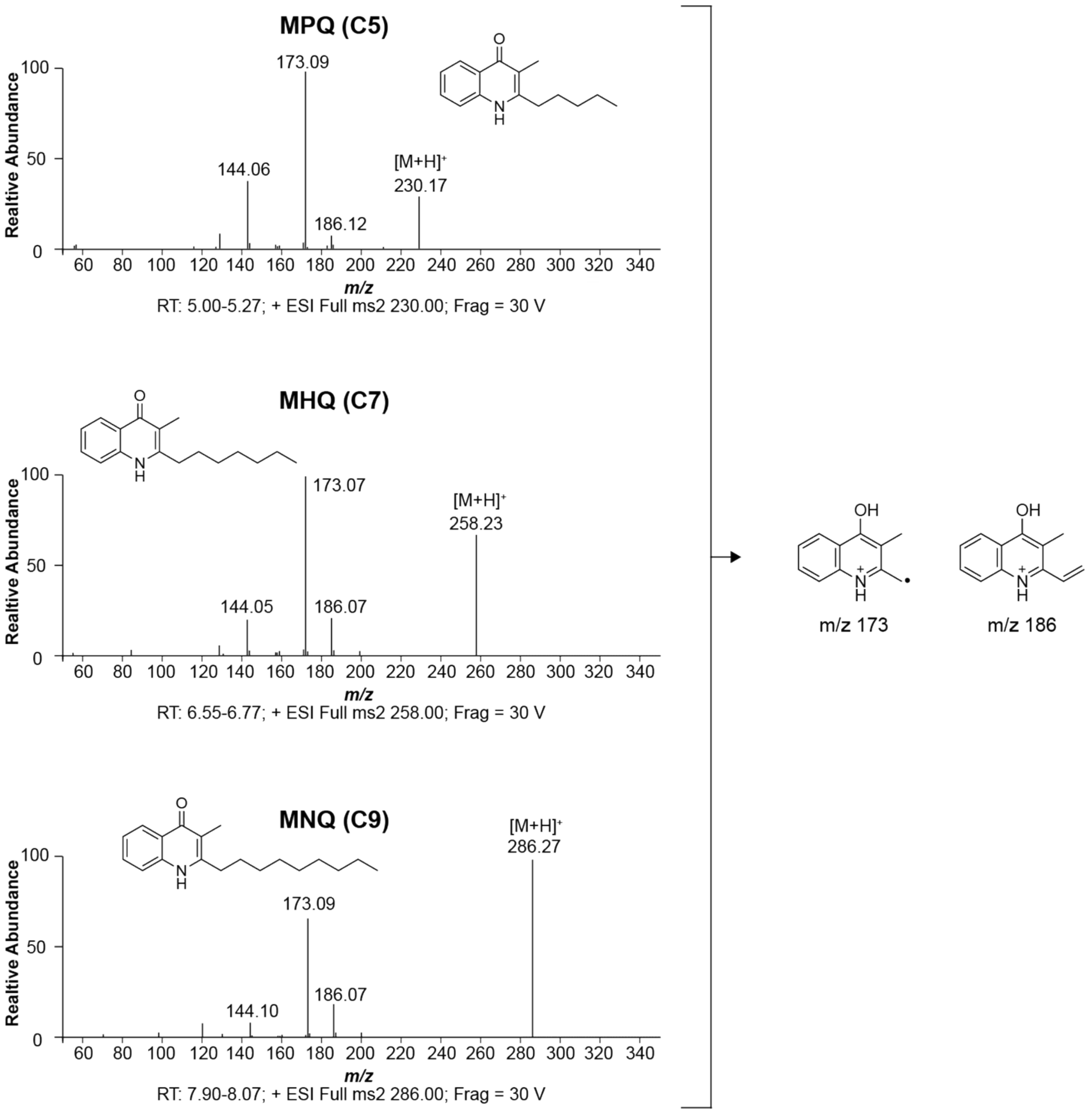

Supporting Figure S8. Fragmentation of 2-alkylquinolones (MAQs) containing a saturated methyl chain produced by *Burkholderia ambifaria* AMMD.

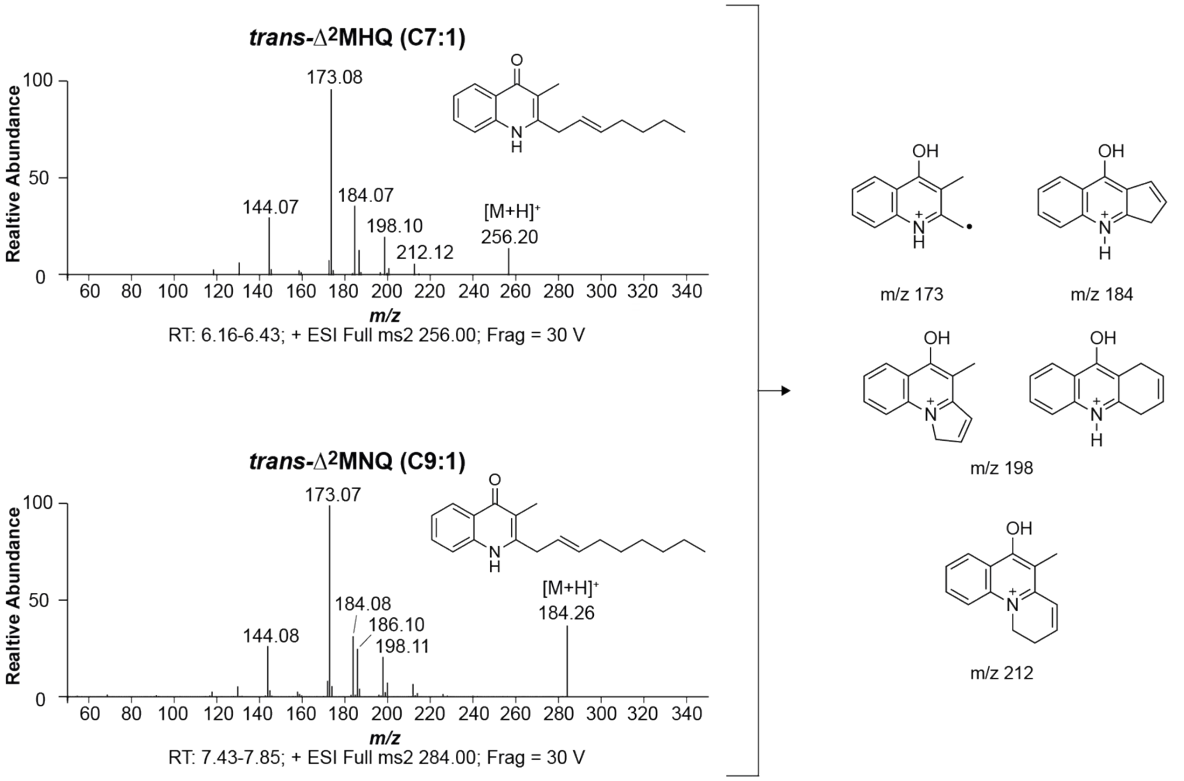

Supporting Figure S9. Fragmentation of 2-alkylquinolones (MAQs) containing an unsaturated methyl chain produced by *Burkholderia ambifaria* AMMD.

A)

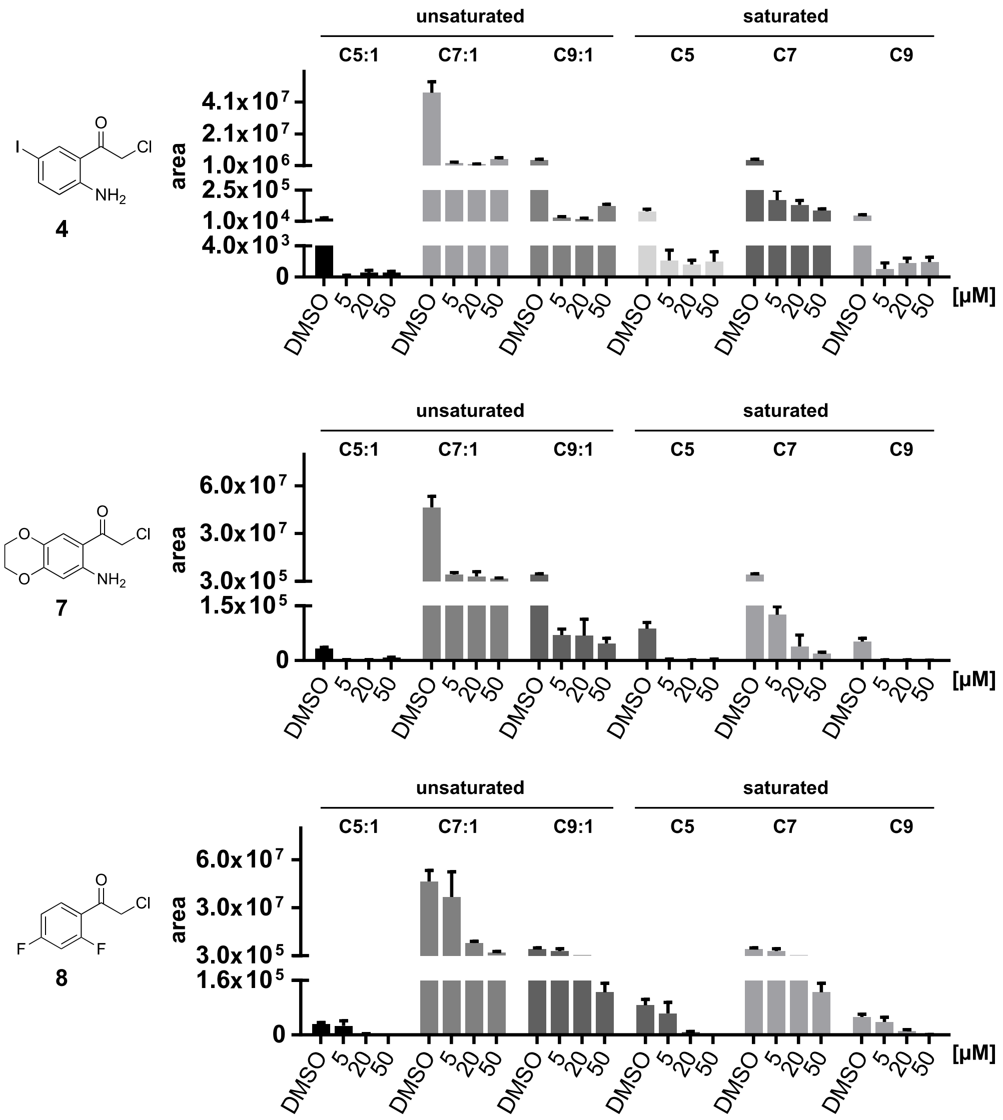

B)

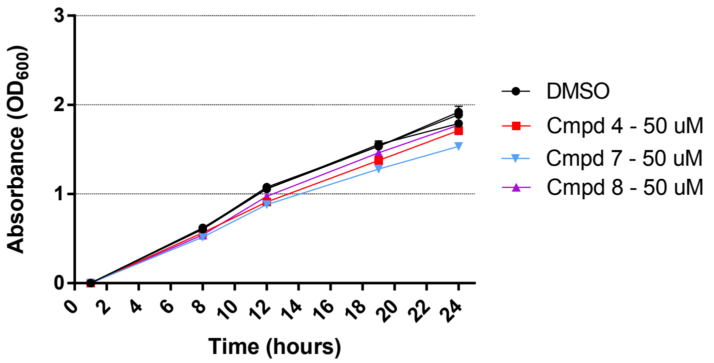

Supporting Figure S10: **A)** Integrated area of mass transitions of selected MAQs detected in supernatants of *B. ambifaria* AMMD after inhibitor treatment. **B)** Bacterial growth during experiment. Cultures were treated with 5 µM, 20 µM and 50 µM of inhibitors for 24 h. Experiments were performed in 3 biological replicates.

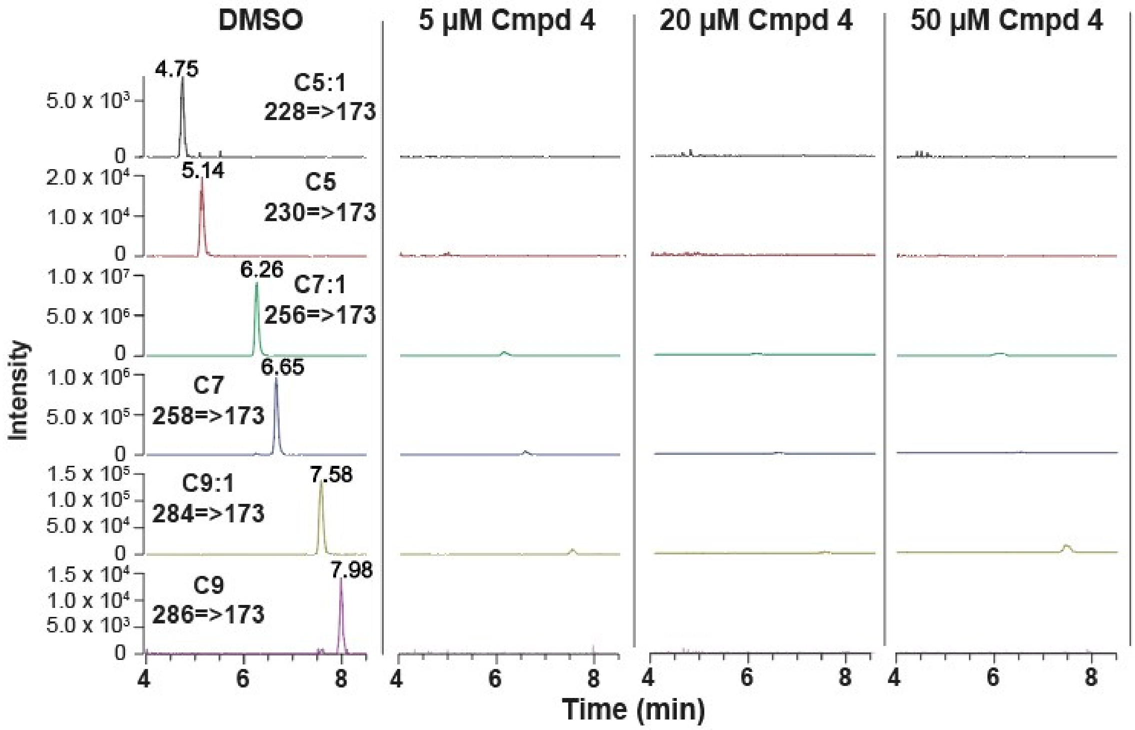

Supporting Figure S11: Recorded LC-MS/MS mass transitions of extracts of *B. ambifaria* AMMD culture supernatants. Cultures were treated with 5 µM, 20 µM and 50 µM of inhibitor **4** for 24 h. Experiments were performed in 3 biological replicates.

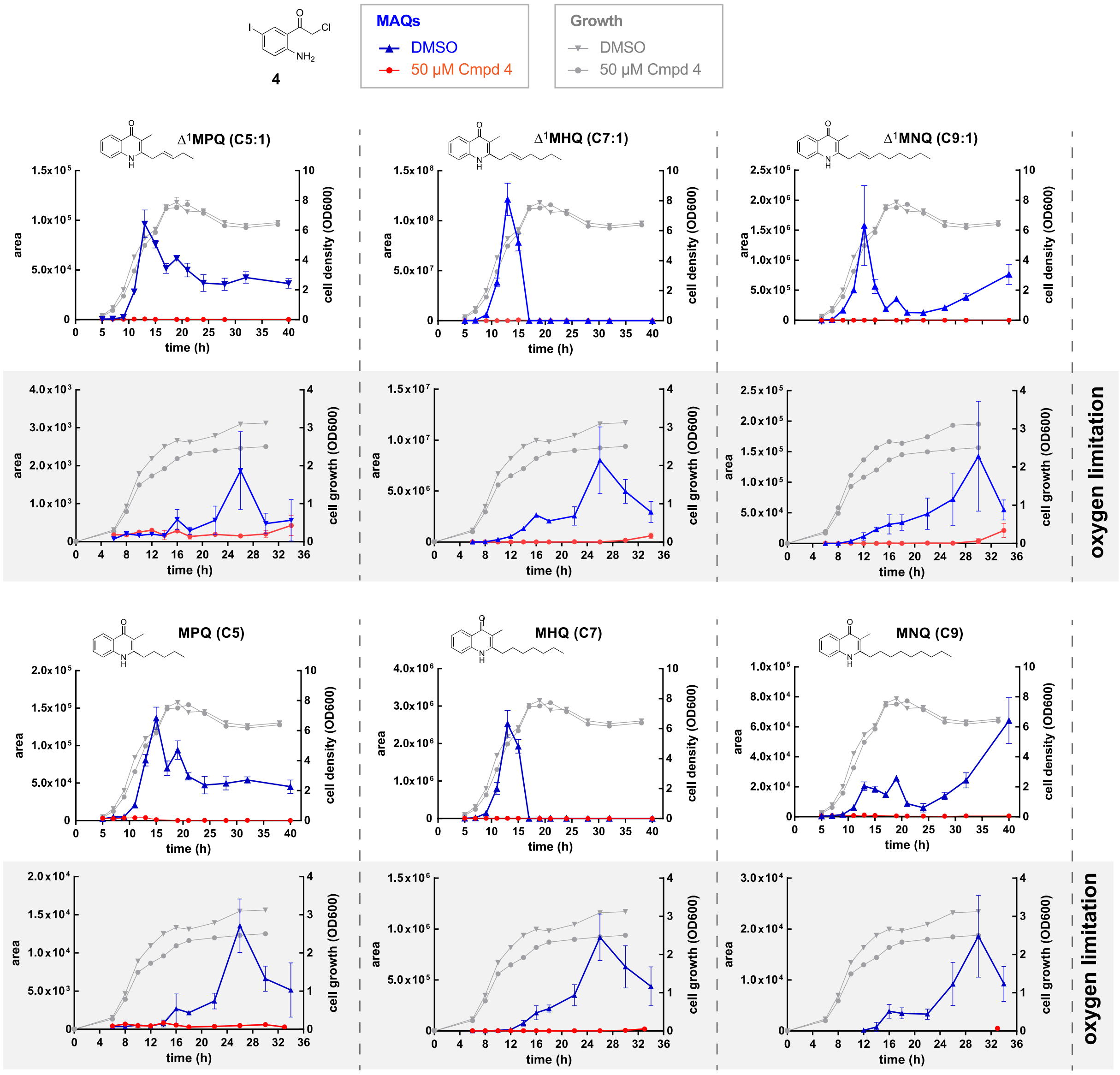

Supporting Figure S12: Time-dependent growth and MAQ production of *B. ambifaria* AMMD.

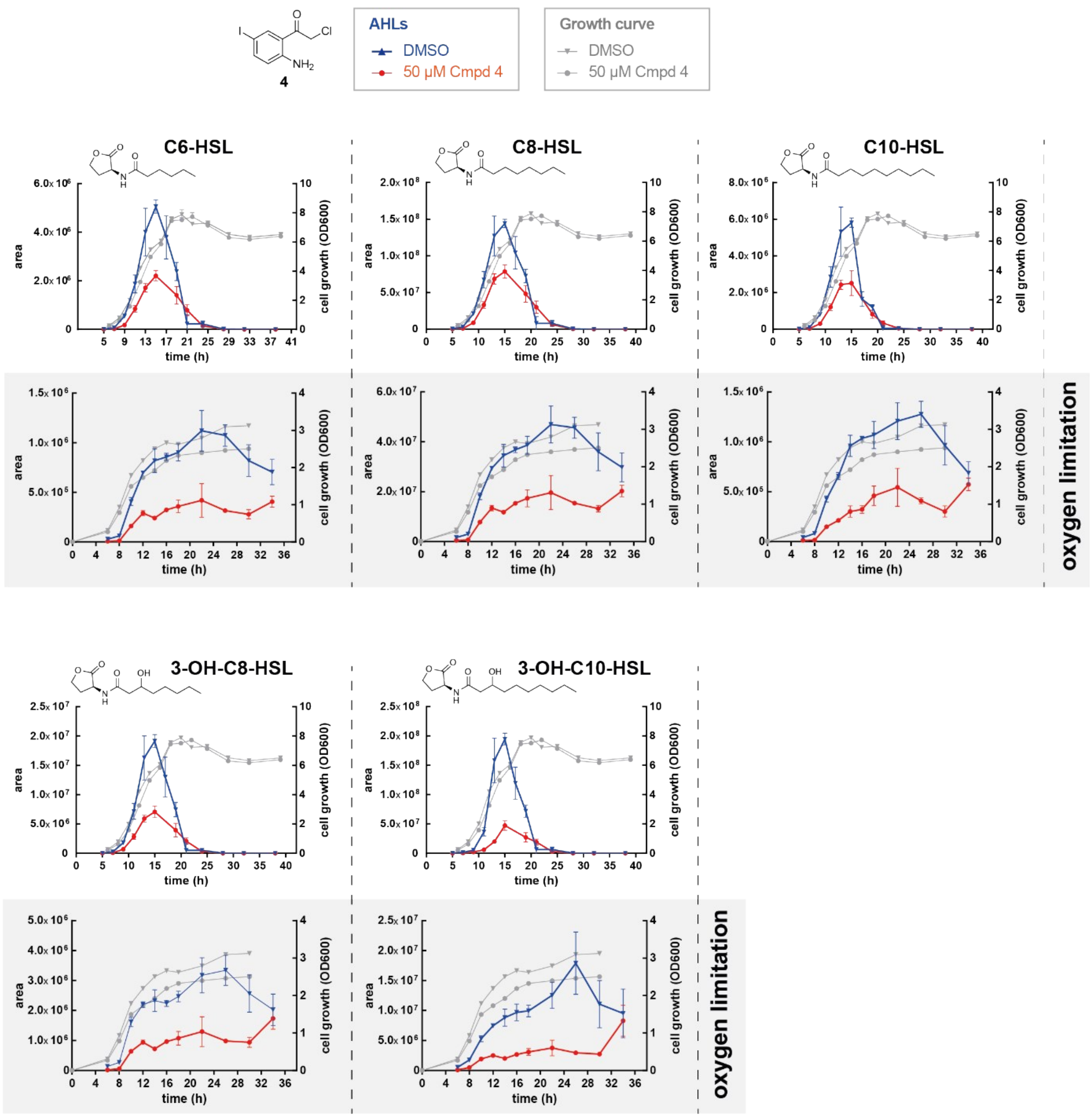

Supporting Figure S13: Time-dependent growth and AHL production of *B. ambifaria* AMMD.

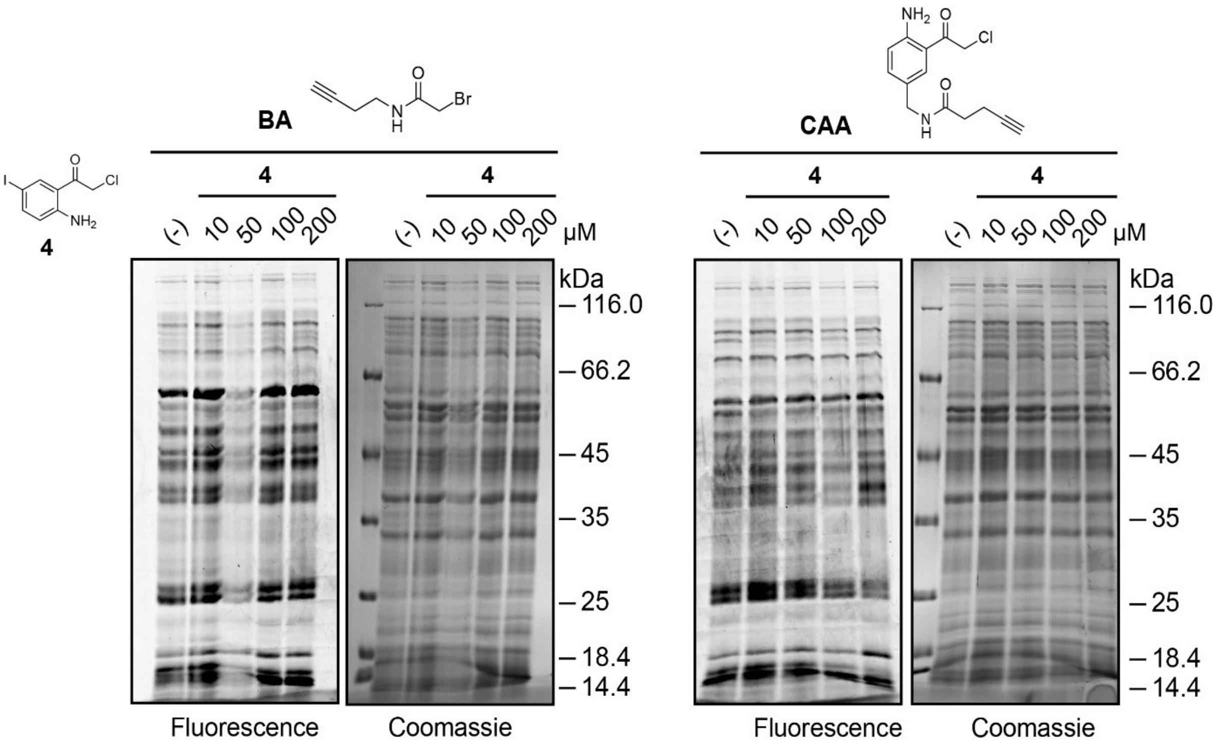

Supporting Figure S14: SDS-gel analysis of competitive *in vitro* labelling of *B. ambifaria* AMMD lysates with probes **BA** and **CAA** (20 µM final probe concentration) after pre-treatment of live cells of *B. ambifaria* AMMD with compound **4**.

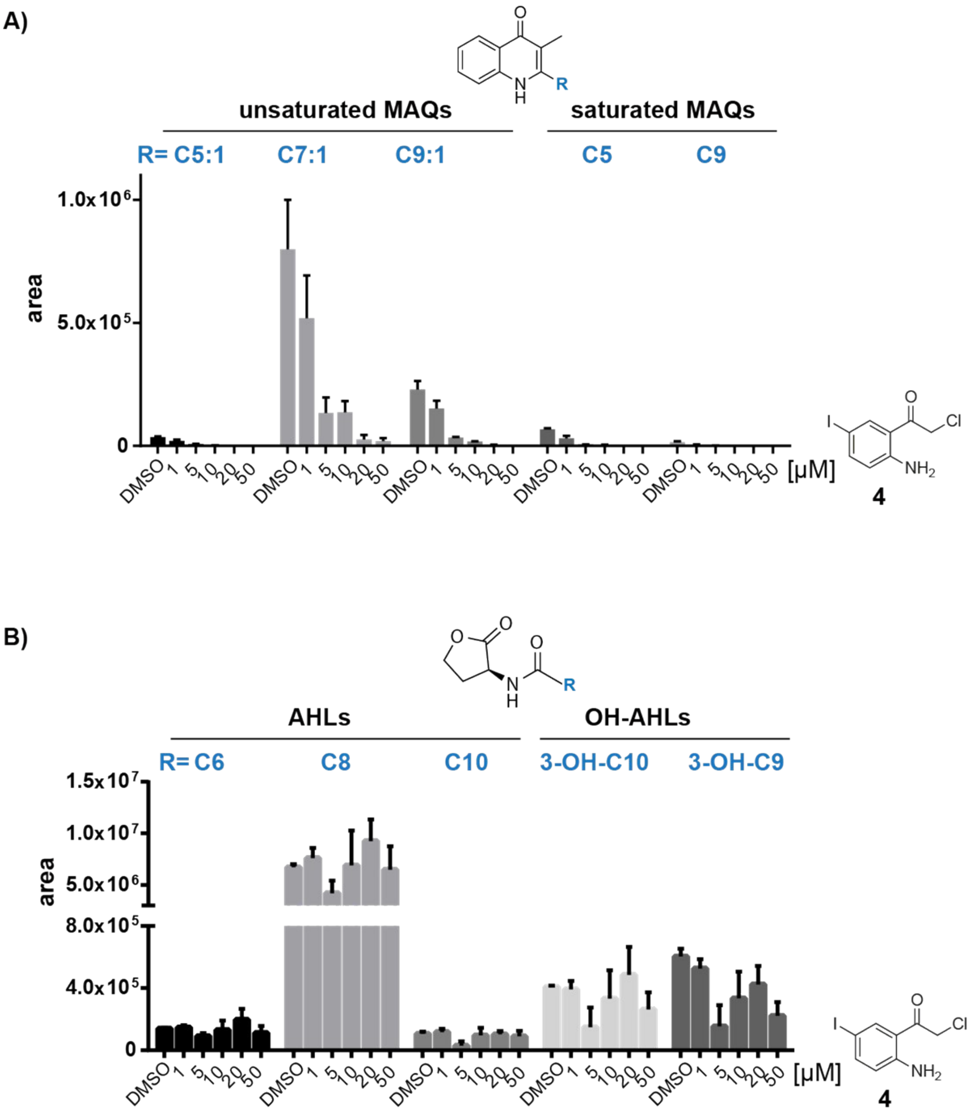

Supporting Figure S15: Integrated area of mass transitions of selected MAQs and AHLs detected in supernatants of *B. ambifaria* AMMD after inhibitor treatment. Cultures were treated with DMSO vehicle, 1 µM, 5 µM, 10 µM, 20 µM and 50 µM of inhibitor **4** for 11 h. Experiments were performed in 3 biological replicates.

a)

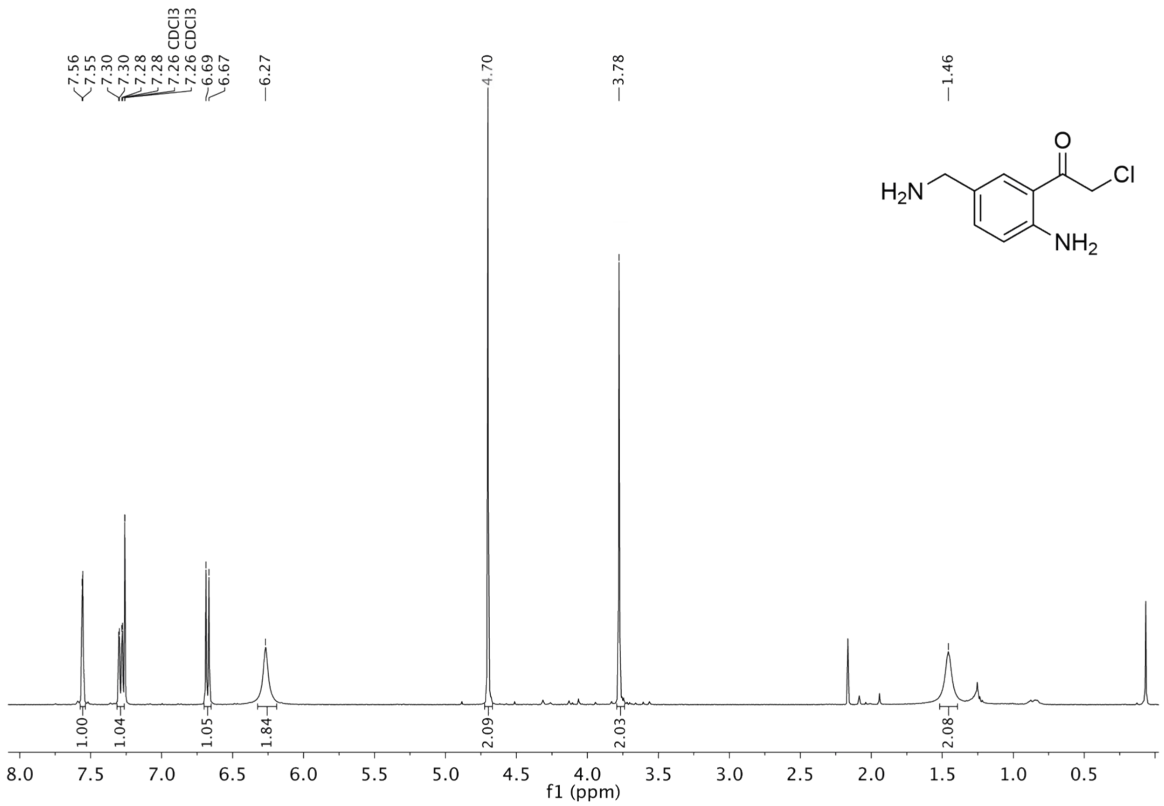

b)

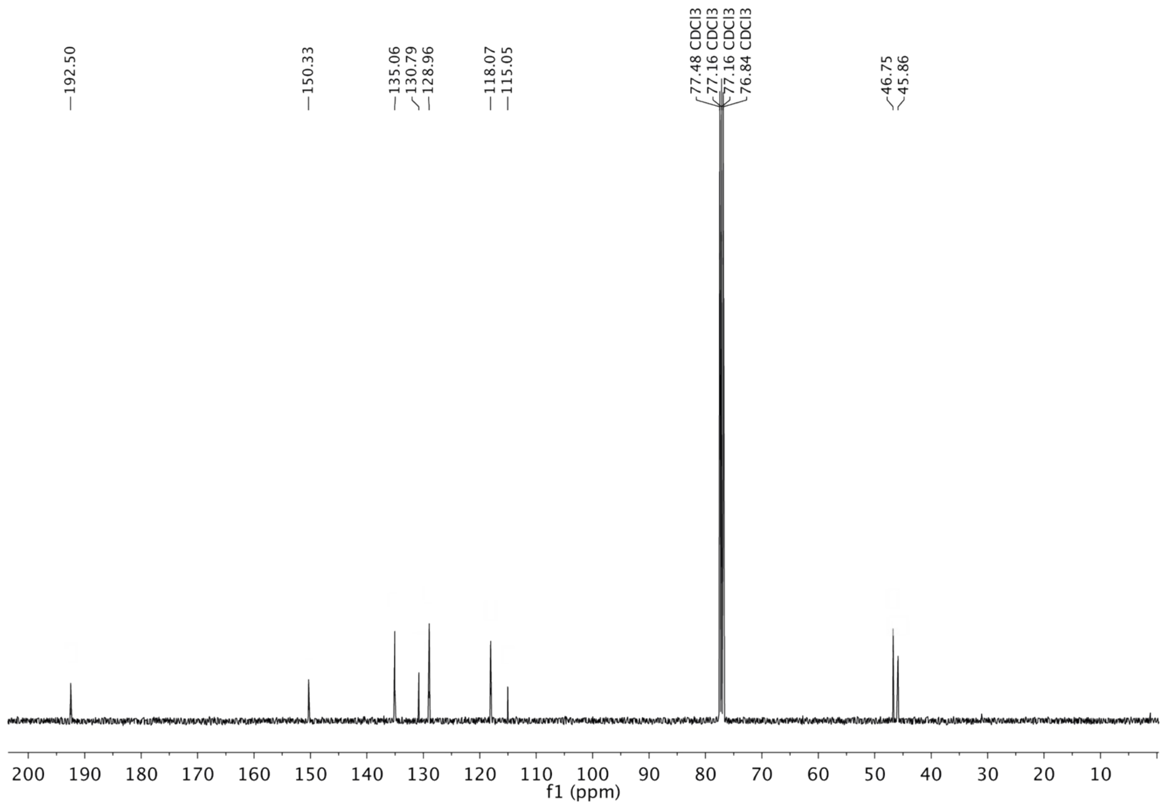

Supporting Figure S16. ^{1}H (a) and ^{13}C (b) spectra of Cmpd **2** in $CDCl_3$

a)

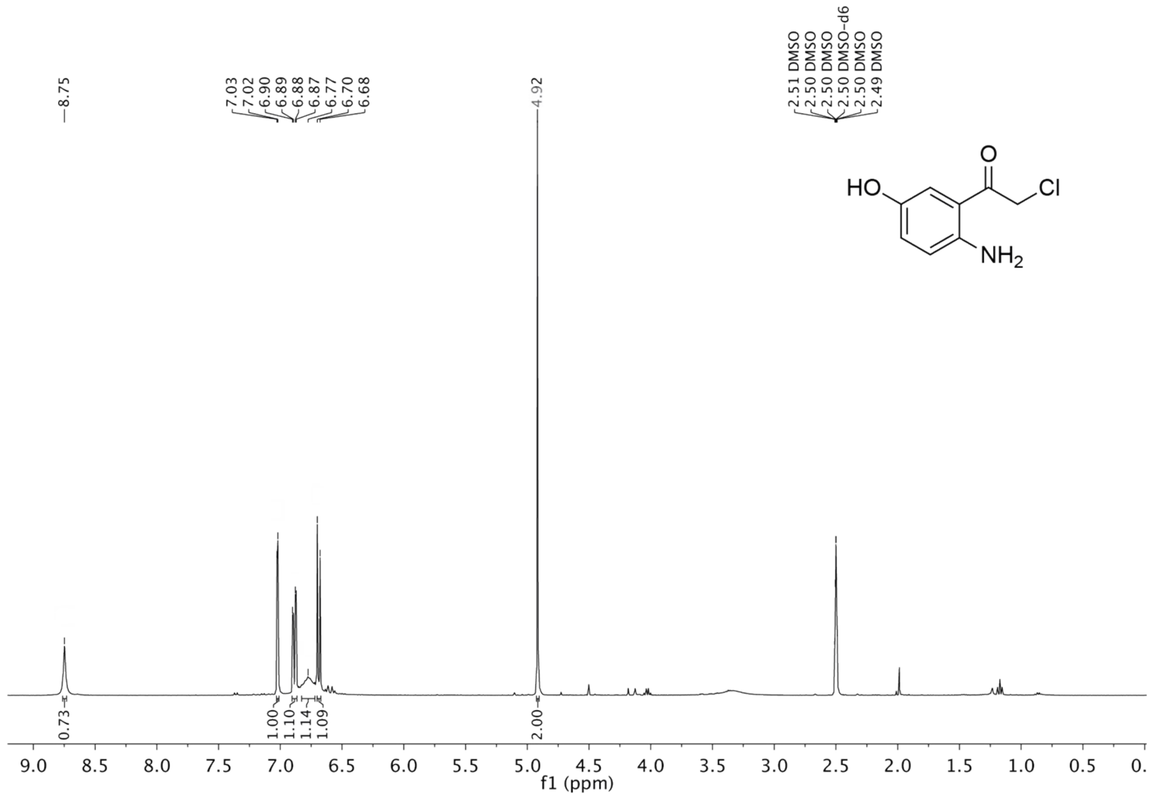

b)

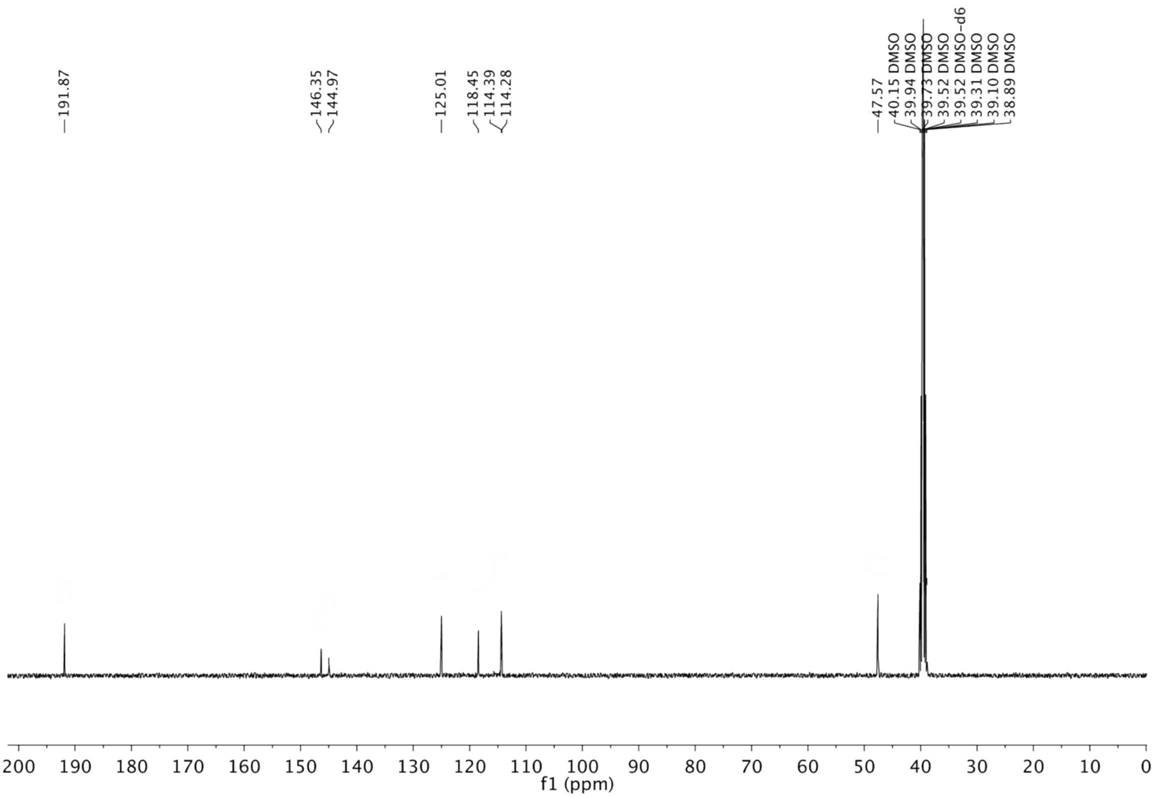

Supporting Figure S17. ^{1}H (a) and ^{13}C (b) spectra of Cmpd **3** in MeOD.

a)

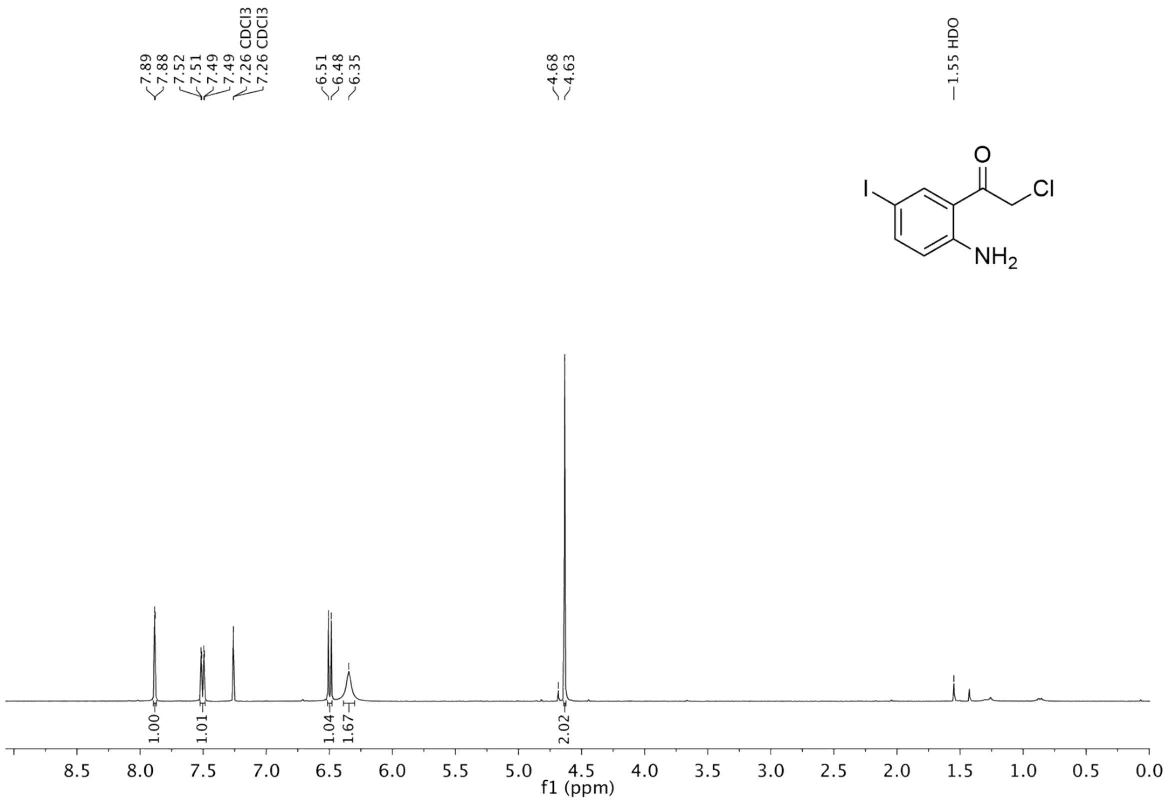

b)

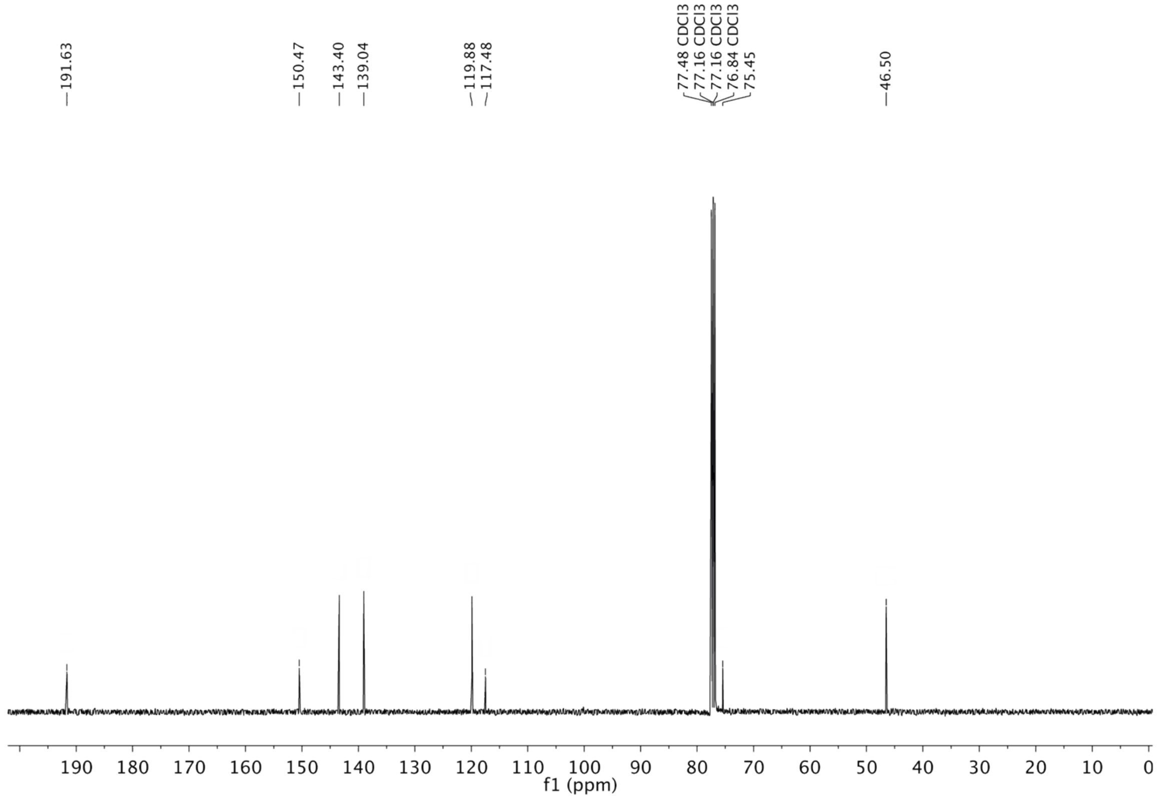

Supporting Figure S18. ^{1}H (a) and ^{13}C (b) spectra of Cmpd **4** in MeOD.

a)

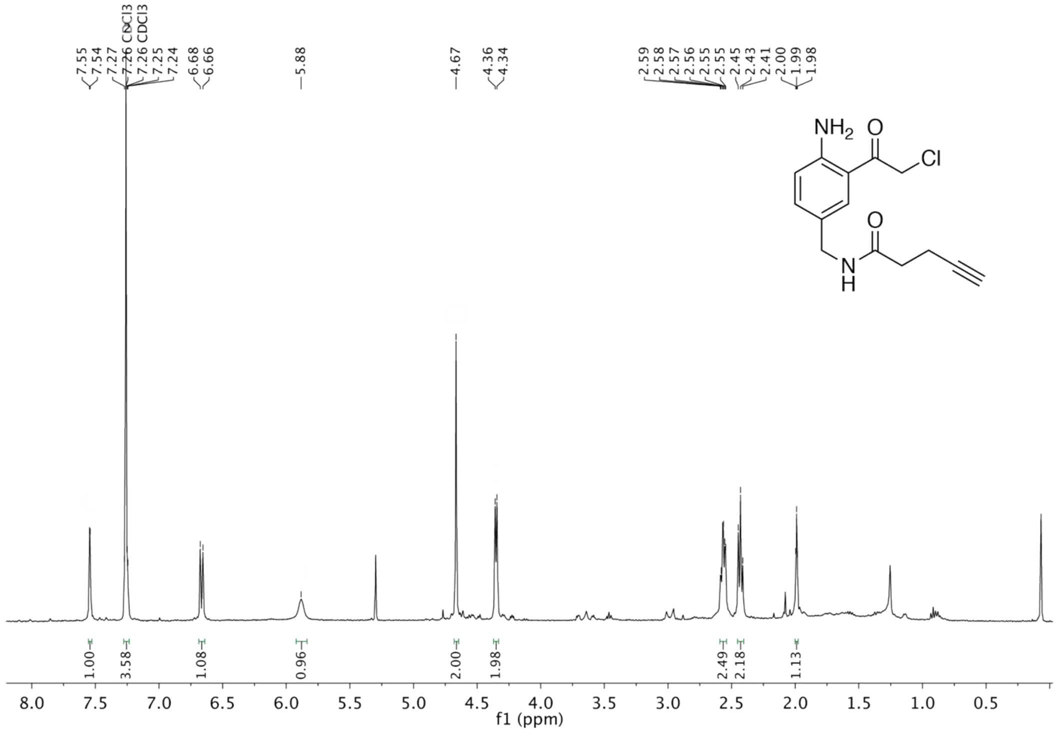

b)

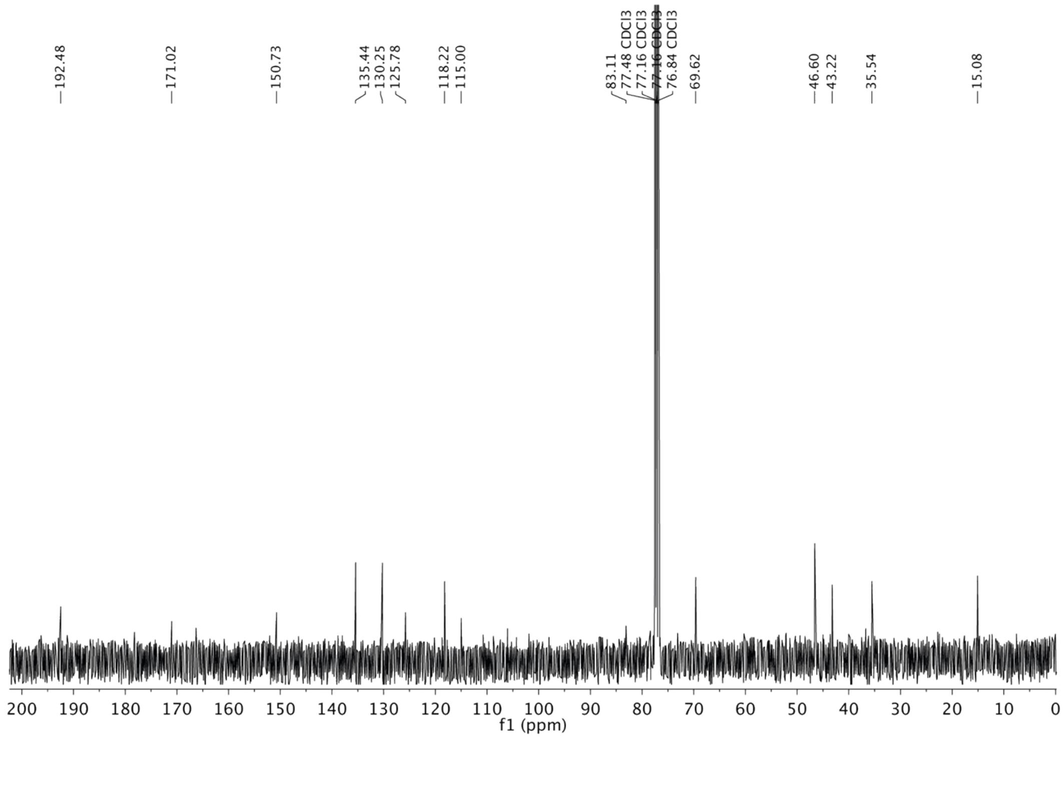

Supporting Figure S19. ^{1}H (a) and ^{13}C (b) spectra of probe **CAA** in $CDCl_3$.

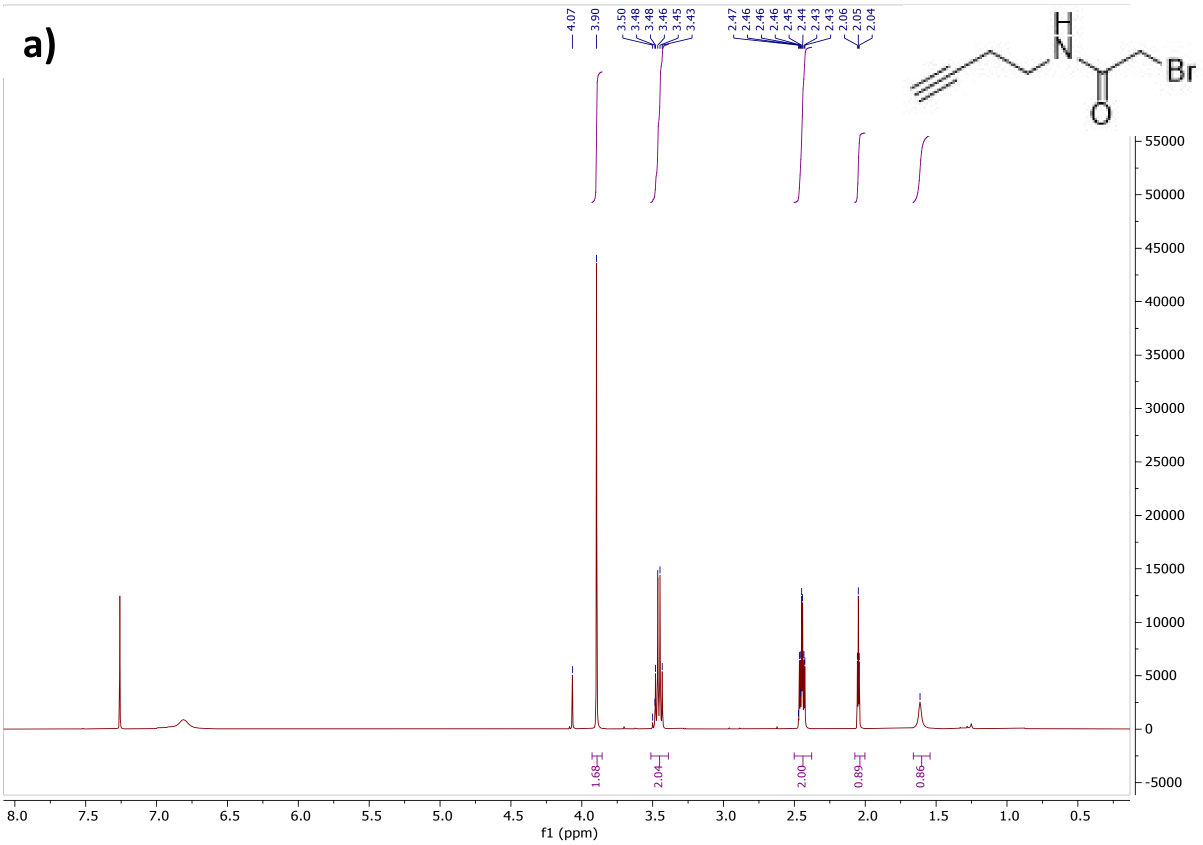

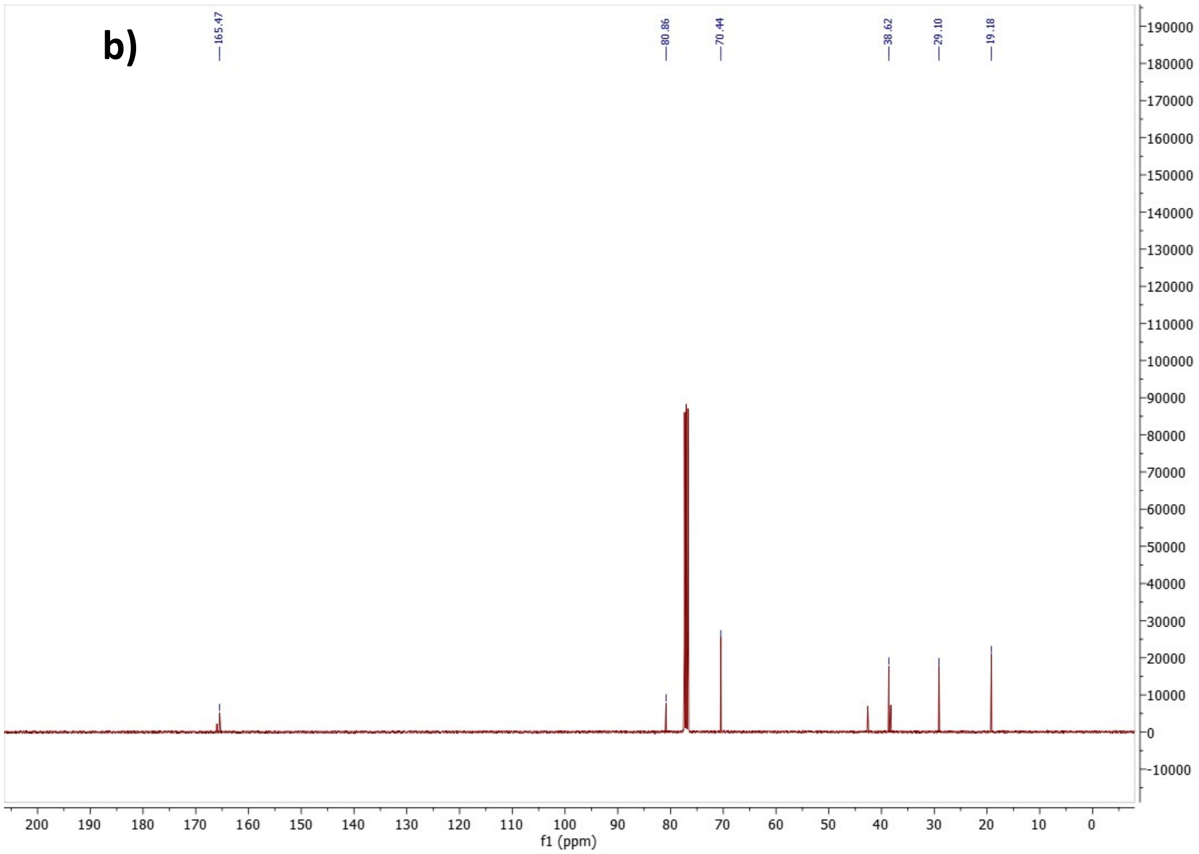

Supporting Figure S20. ^{1}H (a) and ^{13}C (b) spectra of probe **BA** in $CDCl_3$.

a)

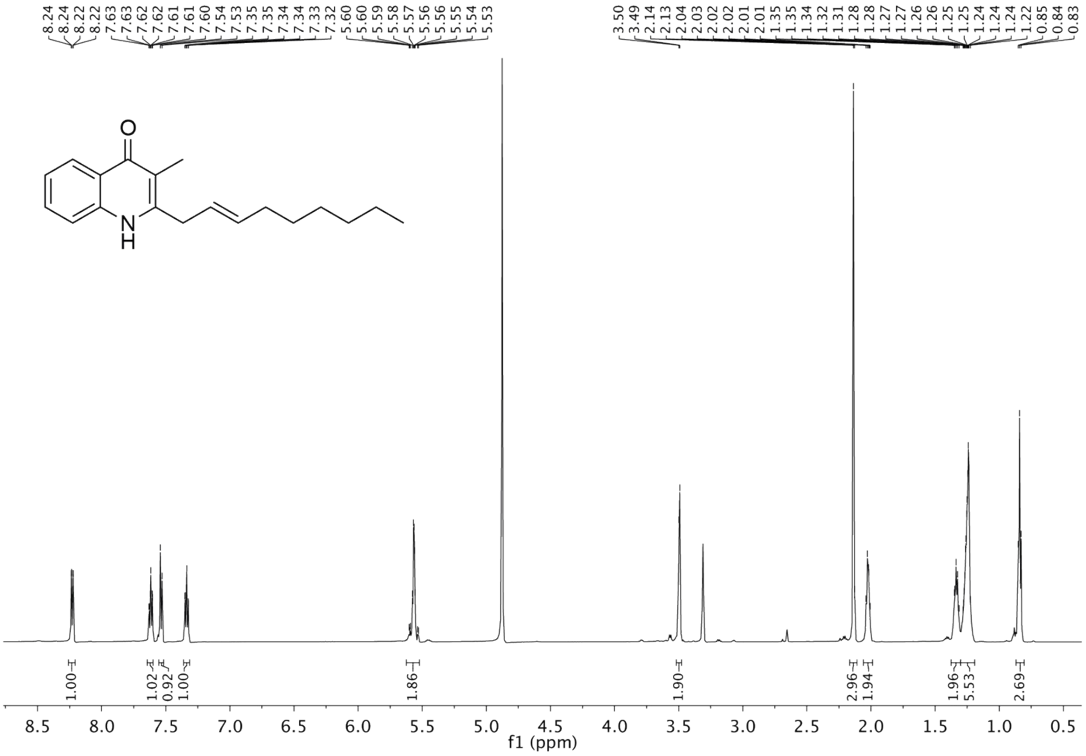

b)

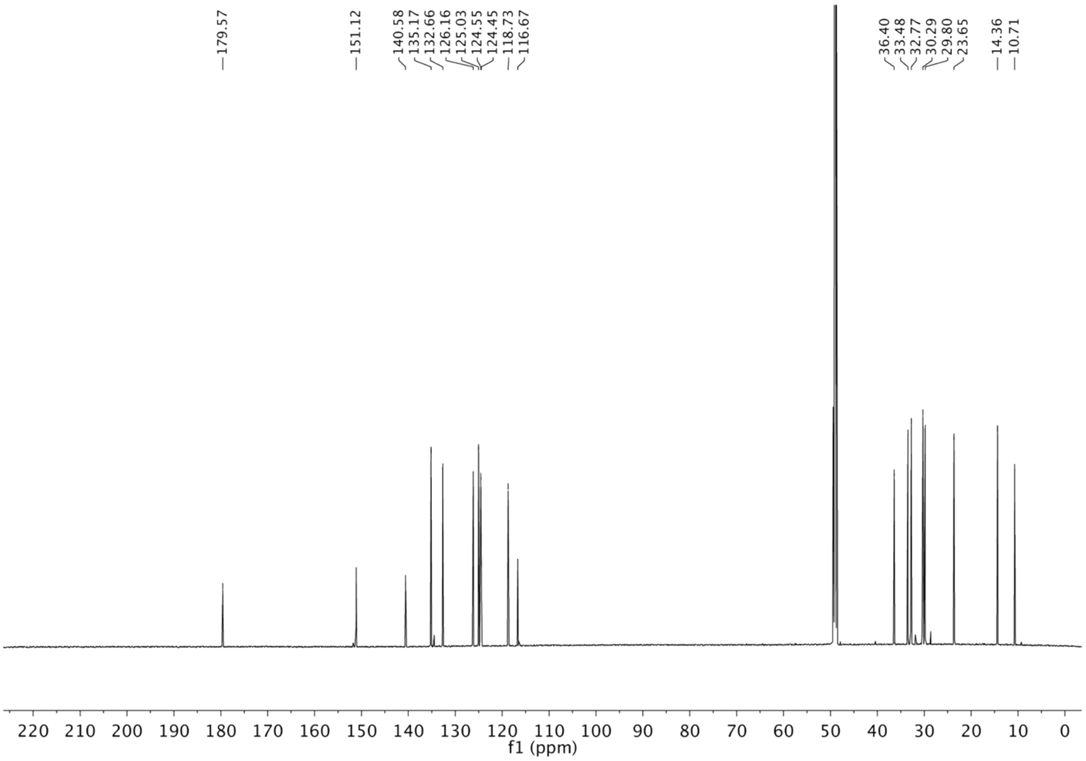

Supporting Figure S21. ^{1}H (a) and ^{13}C (b) spectra of Δ^{2}MNQ in MeOD isolated from *Burkholderia thailandensis*.

References

1. Szamosvári, D.; Prothiwa, M.; Dieterich, C. L.; Böttcher, T., Profiling structural diversity and activity of 2-alkyl-4(1H)-quinolone N-oxides of Pseudomonas and Burkholderia. *Chem Commun (Camb)* **2020,** *56* (47), 6328-6331.
2. Wager, C. A. B.; Miller, S. A., Two robust, efficient syntheses of [phenyl ring-U-14 C]indole through use of [phenyl ring-U-C-14]aniline. *J Labelled Compd Rad* **2006,** *49* (7), 615-622.
3. Prothiwa, M.; Englmaier, F.; Böttcher, T., Competitive Live-Cell Profiling Strategy for Discovering Inhibitors of the Quinolone Biosynthesis of Pseudomonas aeruginosa. *Journal of the American Chemical Society* **2018,** *140* (43), 14019-14023.
